# Transformed Corrugated Shell Units Used as a Material Determining Unconventional Forms of Complex Building Structures

**DOI:** 10.3390/ma14092402

**Published:** 2021-05-05

**Authors:** Jacek Abramczyk

**Affiliations:** Department of Architectural Design and Engineering Graphics, Rzeszow University of Technology, Al. Powstańców Warszawy 12, 35-959 Rzeszów, Poland; jacabram@prz.edu.pl

**Keywords:** systems of planes, parametric polyhedral networks, control tetrahedra, division coefficient method, corrugated shell roof units, complex substitute material, engineering computer models, free-form buildings

## Abstract

This article is an insight into interdisciplinary topics in the field of civil engineering, morphology, architecture, mechanics, and computer programming. A novel method for shaping unconventional complex roofs in which regular folded units transformed into various shells are used as a complex substitute material is proposed. The original method’s algorithm for building systems of planes defining diversified polyhedral networks in the three-dimensional space by means of division coefficients of the subsequently determined vertices is presented. The algorithm is based on the proportions between the lengths of the edges of the reference network, the location and shape of the ruled shell units included in the designed complex roof structure, so it is intuitive. The shell units are made up of nominally flat folded sheets transformed effectively into shell forms whose static-strength properties are controlled by geometric quantities characteristic of ruled surfaces. The presented original approach to the shaping of the shell roof structures determining specific complex building forms allows us to go beyond the limitations related to the orthotropic structure of the folded roof sheeting and the shape transformations.

## 1. Introduction

**Figure 1:**
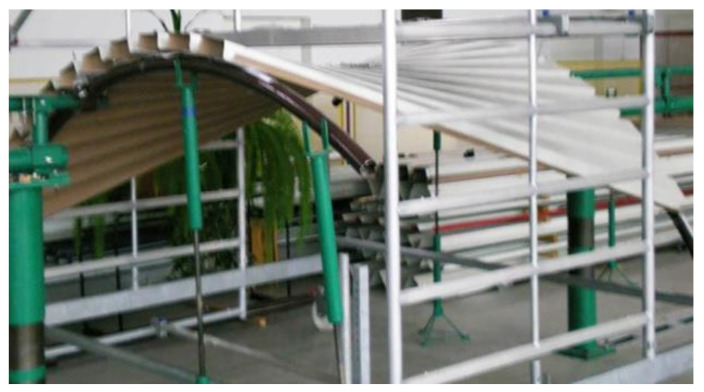
Experimental folded shell sheeting supported by two curvilinear skew directrices.

Since the transverse flexural stiffness and torsional stiffness of a nominally flat thin-walled steel sheet of open profile and folded in one direction are small, then a small load applied perpendicularly to the neutral surface of the sheet causes a significant initial shape change. The loaded sheets connected by their longitudinal edges into a nominally flat single strip are spread on at least two mutually skew directrices to change their forms from flat into ruled shells, depending on the shape and the mutual position of the directrices [1] (Figure 1).

In order to reduce the initial stresses resulting from the aforementioned shape transformations, each fold tends to balance the tensile stresses appearing at both fold’s transverse ends, with the compressive stresses appearing at a half along the fold’s length. If a freedom of the fold’s changes manifesting especially in the fold’s width increments is ensured during spreading and fixing all folds to the roof directrices, then the folds tend to obtain such shell forms that their longitudinal axes remain straight lines and their contraction appears at a half-way along the length of each shell fold. The geometry of all subsequent cross-sections of each transformed fold variously changes along the fold’s length, so that each fold reaches the maximum contraction and the maximum height at a half along its length.

**Figure 2:**
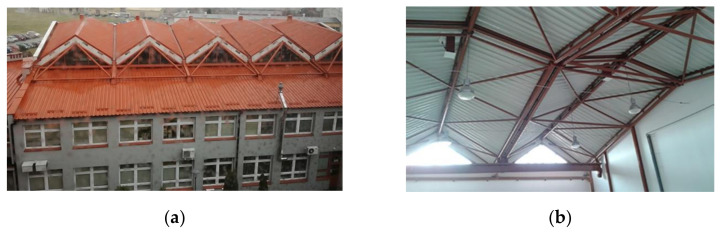
Complex shell structure roofing an experimental hall: (**a**) an outside view; (**b**) an inside view.

Since the length of the thin-walled steel sheets is relatively small and the aforementioned geometric and structural limitations of the effectively transformed sheets are very important boundary conditions, it is impossible to obtain a single smooth shell roof of a medium span [1,2] (Figure 2). Therefore, the complete transformed shell sectors are joined by means of their transverse edges into ribbed structures, so that the shape of each shell sector is characterized by a contraction passing through the halves of all folds at their length to optimize the initial stresses [3].

**Figure 3:**
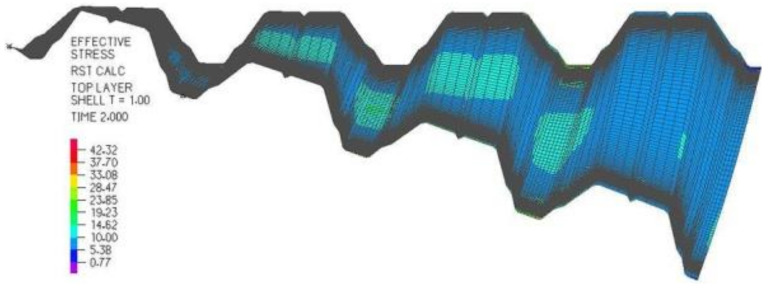
The accurate computational mechanical model of an elastically transformed sheet and the graphical expression of the “effective” stresses in MPa on its top surface.

For scientific research, experimental tests and computer simulations are carried out by Abramczyk [1,4] (Figure 3), where the transformed experimental folded sheeting is accurately modeled with computational thin-walled folded sheeting. The geometry, strength and stability of these models are analyzed by Abramczyk [5], using advanced dynamic incremental non-linear methods described by Bathe [6].

## 2. Critical Analysis of the Present Knowledge

The complete thin-walled folded shells transformed into central sectors of hyperbolic paraboloids were investigated by many researchers. The main geometric and mechanical properties of folded sheeting deformed into corrugated hyperbolic paraboloid shells were presented by Nilson [7]. The Winter’s team [8] confirmed Nilson’s results and expanded on the scope of the performed tests and analysis. Parker analyzed structures composed of a few quarters of right hyperbolic paraboloids made of two layers of sheets located orthogonally in the same shell.

McDermott [9] described the behaviour of a central sector of a folded steel hyperbolic paraboloid stiffened with a circumferential frame. Parallel studies related to the static-strength work single and complex hyperbolic paraboloid shells were conducted by Egger et al. [10] based on the conventional analysis and analytical calculations of strength and critical loads. Gergely et al. [11] carried out a detailed analysis of the static-strength work of the single and complex profiled hyperbolic paraboloid shells composed of two orthogonal layers, which enables these research studies to analyze the shells as isotropic.

**Figure 4:**
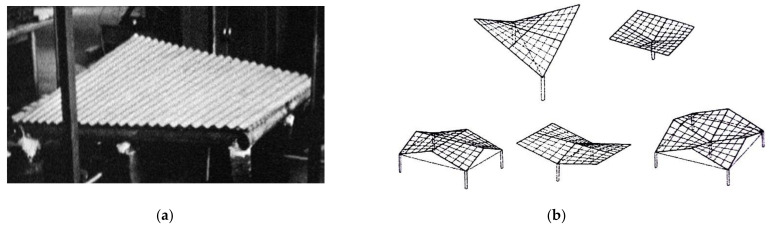
Two symmetric experimental hyperbolic paraboloid shells: (**a**) a complete shell; (**b**) umbrella structures of four quarters.

Gioncu and Petcu [12] developed the novel HYPBUCK computer program for calculating the critical loads. Davis and Bryan [13] pointed out the most important geometric and mechanical characteristics of the transformed shell folds, to make it easier for the designer to shape the transformed shells. Finally, they stated that theoretically it is possible to shape many different types of the transformed folded shell sheeting. Practically, it is possible to build only cylindrical and shallow corrugated hyperbolic paraboloid roofs due to the available engineering technology. In summary, all the investigated shells underwent forced shape transformations, causing relatively big initial stresses, because the fold’s longitudinal axes were adapted to the selected rulings of the adopted hyperbolic paraboloids. Therefore, the initial stresses had to be restricted, for example, by limiting the transformation degree. As a result, only shallow hyperbolic-paraboloid shells called hypars can be created (Figure 4).

Biswas and Iffland [14] presented a few concepts of shaping continuous regular roof structures composed of many identical hyperbolic paraboloid segments made up of transformed folded steel sheets. They arranged many complete shell sectors on a sphere to increase the span of the designed free form building.

**Figure 5:**
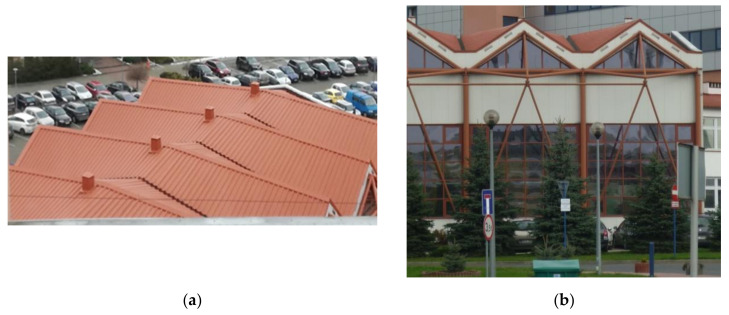
The external views of two opposite elevations of the experimental hall at a university roofed with the shell structure: (**a**) the roof structure; (**b**) the south side of the entire building.

The thin-walled corrugated shell steel roofs transformed freely are shaped by Reichhart [15] and simply modeled with various right hyperbolic paraboloids (Figure 5) and other deeply ruled surfaces [16]. For geometric engineering developments, each shell sheeting can be modeled with a smooth sector of a ruled undevelopable surface called a warped surface [1] (Figure 1) including hyperbolic paraboloid [17] (Figure 2).

In order to create a method for shaping corrugated shells transformed rationally, Abramczyk has proposed a condition requiring the contraction of a single shell to pass halfway along the length of each shell fold [1] (Figure 3). Abramczyk’s method employs some specific geometric properties of regular warped surfaces, primarily their lines of striction. The second condition utilized by Abramczyk relates to determining certain surface areas of the created smooth shell models corresponding to the compressing and stretching zones of each transformed fold [3]. These two conditions are based on the results of his experimental tests and computer simulations (Figure 1 and Figure 3).

The quarters of the hyperbolic paraboloid shells are often arranged symmetrically in different configurations to increase the expected roof spans. In order to create diversified free form building structures roofed with complex corrugated shells characterized by medium spans, Prokopska and Abramczyk [18,19] analyzed the so-called reference tetrahedrons to model a complete free form covered with oblique plane elevations and roofed with many transformed complete corrugated shell sectors arranged regularly in the three dimensional space [20].

**Figure 6:**
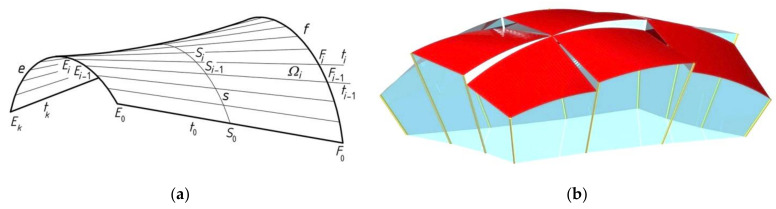
(**a**) A simplified geometric smooth model of a complete corrugated shell sheeting; (**b**) A simplified geometric model of a building free form roofed with complex corrugated shell sheeting structure.

Each single shell segment is modeled by Abramczyk for the engineering developments [1] with a sector of a warped surface [21] (Figure 6a), limited by a closed spatial line composed of four segments contained in the planes of a so-called reference network [22]. The planes of the reference network divide the complete shell segments of the designed shell roof structure. They are helpful in defining roof directrices (Figure 6b). In the present article, all directrices *e* and *f* are straight sections.

**Figure 7:**
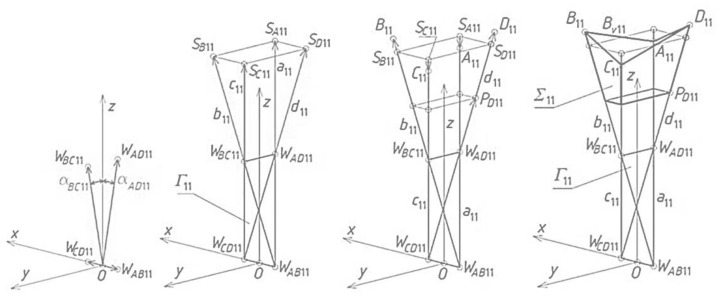
Stiff motions: translations and rotations for creating meshes *Γ*_11_, *B_v_*_11_ and *∑*_11_.

Abramczyk [23] developed a method for shaping axis symmetric polyhedral networks based on rigid motions of all vertices of these networks (Figure 7). Four adjacent planes of each reference network limit one tetrahedral mesh. A parameterization of the reference networks enables one to shape various configurations of attractive complex building free forms and innovative structural systems intended for these free forms by means of computer technique.

The above-mentioned method allows one to transform the shapes of various types of building structures proposed by Abdel and Mungan [24] and Saitoh [25]. Free-form shell bar structures are presented by Obrębski [26]. Structural bar systems for several types of the corrugated roofs were developed by Rębielak [27]. Free-form buildings and their constructions were analyzed by Reichhart [28] and Wei-Wen [29]. Samyn suggests interesting aluminium hypar forms [30].

The most general method of architectural shaping of polyhedral shell structures was developed by Pottmann et al. [31,32]. The designed geometry of these structures does not result from the orthotropic properties, rectangular shapes of the folded sheets and the shape transformations of the designed roof shells. 

The folded steel sheeting transformed elastically or plastically can be used as a structural shell unit supporting appropriate roof covers [33]. The classical theory of elasticity proposed by Green and Lindsay has been extended by Marin et al. [34] to cover the theory of thermoelasticity for dipolar bodies. A specific method of unique solution of a mixed problem in the dynamical case is proposed using a reciprocal theorem [35]. These fundamental results were obtained under not very restrictive conditions.

## 3. The Aim and the Scope of the Research

The aim of this article is to present a novel method for shaping unconventional complex shell roof structures whose various complete folded shell segments are used as a complex substitute material. The unit segments of a similar ruled form are located in the three-dimensional space in an orderly and effective manner using a reference regular surface and an innovative regular polyhedral network whose straight edges are normal to this reference surface.

The presented parametric development of the specific polyhedral reference networks enables one to computationally shape diversified unconventional complex building free forms roofed with regular structures composed of many different shell segments made up of nominally flat thin-walled steel folded sheets transformed into various corrugated shell shapes. Simplified geometric models of the complete roof shells created by means of the method as smooth sectors of warped surfaces are sufficient for most engineering developments. These models can be determined by means of the reference and eaves networks investigated in the present article. 

The method’s algorithm must be supported by computer technology due to the accessibility of the method for designers. This is why a few novel computer applications were written in one of the CAD programming languages. The important feature of the algorithm is that the proposed reference networks enable an intuitive and automatic modification of the obtained results to adapt these networks to the geometric and static-strength characteristics of the transformed folded sheets and the required geometric boundary conditions. These conditions are related to some initially adopted proportions between the dimensions of all main elements of the designed building and the shape and mutual position of all pairs of the designed roof directrices.

The description and the computer implementation of the presented algorithm, helpful in searching for possible and acceptable parametric configurations and modifications of the reference networks, have led to rational solutions related to: (1) attractive forms of the designed complex building forms, in particular multi-plane folded facades and multi- segment shell roof sheeting, (2) satisfactory stability and the smallest possible effort causing by the investigated shape transformations, and (3) rationality of shaping of bar structural systems intended for the complex building forms.

## 4. The Method’s Concept

The process of shaping the built environment by means of diverse unconventional building forms requires one to solve many interdisciplinary issues in the field of town planning, architecture, morphology, civil engineering, constructions and computer support. Each issue is considered at different levels of accuracy, which further complicates the process of conducting the relevant analysis. The complex design process must therefore be divided into many steps related to the function, arrangement and type of the components of the building shaped, as well as the level of the modeling accuracy and execution of these elements.

The relation between the formation of the urban space and the social experience of the human self must be taken into account [36]. The formation of the space investigating its physical form and cultural patterns into a whole spatial system is very important.

The design syntax of urban greenways should also be explicitly discussed. In this way, mathematics-based graph studies to analyze patterns and shapes, photography based thermal, material and morphology studies, and section analyses to make imagery-derived deductions on the design syntax are carried out [37].

The morphological shaping of buildings plays a significant role in the design process, by using features specific for architectural, industrial and structural design [38]. Morphology is a study of the forms taking account of the adopted interrelationships between the function, structure, internal and external texture, static-strength work and comfort conditions ascribed to the designed building object.

In each of the above mentioned aspects of the design process, a strict ordering of the objects characteristic for the considered step must be carried out. Therefore, according to the deliberated step, the general model is divided into many consistent individual elements and then into sub-elements arranged in a strictly defined order. These elements are adapted to the utilized concept, in accordance with the production technology and the assembly technique.

Thorough research and tests allow one to create appropriate geometric and static-strength models used in the search and development of the new concepts and techniques. The created unconventional new forms result from various innovative approaches to the shaping and ordering of their individual elements, for example in the three-dimensional Euclidean space.

Systematic morphology is defined by Eekhout as “the study of the system, rules and principles of form has led to the interpretation of the study of the geometry of regular three-dimensional bodies or forms, usually known as polyhedra.” [38]. Analogous systems of planes called polyhedral reference networks are used in the present article. These are sums of many regular tetrahedrons arranged regularly in the three-dimensional space.

Wester defined structural morphology as “the Study of Form” [39]. A detailed description of the issues related to the morphology of the structure is presented by Qingpeng [40]. In this article, the study is limited to the geometric shaping of complex building structures roofed with free-form transformed corrugated shell structures arranged regularly in the three-dimensional space. The arrangement of the complete roof shell sectors is carried out by means of plane systems called polyhedral reference networks. The individual planes of each system separate single roof shells and contain the directrices of these shells. The diversification of the shape and mutual position of each pair of the adjacent directrices enables one to shape various innovative transformed forms of the complete corrugated shells.

The following factors have a decisive impact on the geometric properties of the investigated reference networks: (1) the regularity of the shapes and arrangement of the designed roof structures, (2) the rectangular shapes of the utilized folded sheets connected by their longitudinal edges into a single transformed strip, respectively, (3) the shape and mutual position of the roof directrices supporting each individual transformed shell segment, and (4) multi-wall character of the shaped complex facades. The assumed multi-segment nature of each roof structure *Ω* divided by the specific sets of planes allows all individual shells *Ω_ij_* to be arranged by joining together along their transverse edges in relation to the fold directions. This way of connection results in specific ribbed roof structures.

Two adjacent shells of a roof structure can be connected along common eaves lines, including directrices (Figure 2 and Figure 5), or separated by flat window areas, allowing the sun rays to illuminate the building interior. This method allows the designer to shape freely attractive free forms of the roof structures (Figure 6b). In the last case, the roof directrices of two adjacent shells are contained in the same plane, which forced development of a specific method for creating the special systems of these planes called polyhedral reference networks.

The quadrilateral nature of each closed edge line *B_vij_* limiting each complete transformed roof shell *Ω_ij_* results in that each reference network *Γ* is composed of meshes *Γ_ij_* restricted by tetrads of planes. Due to the rectangular characteristics of the individual sheets and their strips, the meshes are built as specific reference tetrahedrons whose four walls contain four single segments of the edge line *B_vij_* of each single shell *Ω_ij_*. Thus, all *B_vij_* are closed spatial quadrangles whose all four apex angles are close to right angles.

**Figure 8:**
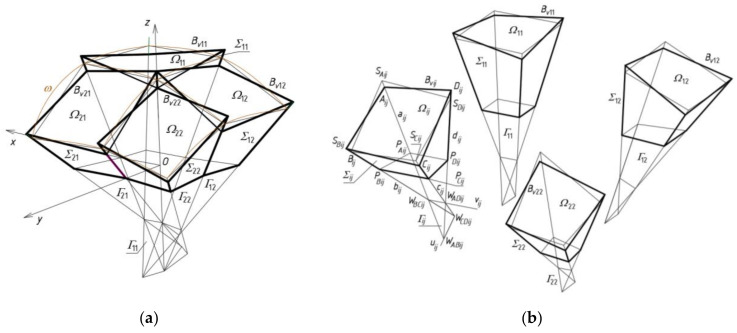
Creating complex building free form by means of a polyhedral reference network consisting of tetrahedral meshes divided by common sides: (**a**) the obtained *Γ* and *∑* after setting up *Γ_ij_* and *∑_ij_*, with each other; (**b**) *Γ_ij_* and *∑_ij_* before setting.

As a rule, the complete shell segments are variously transformed, because they take relatively different forms depending on the shape and mutual position of their roof directrices adapted to the diversified curvature of the whole roof shell structure. Many special regular single shell sectors *Ω_ij_* and complete free forms *∑_ij_* should be located in one polyhedral network mesh *Γ_ij_* (Figure 8a). Thus, the respective façade walls, side edges, roof directrices and eaves segments are included in the tetrad of planes of each *Γ_ij_*. The most general type of the tetrahedral meshes *Γ_ij_* was adopted in the further analysis (Figure 8b). Every two adjacent planes of the same mesh *Γ_ij_* intersect in a side edge *a_ij_*, *b_ij_*, *c_ij_* or *d_ij_*, however, two of its opposite planes intersect in one axis *u_ij_* or *v_ij_*. The side edges and axes are defined by means of four vertices *W_ABij_*, *W_CDij_*, *W_ADij_* and *W_BCij_*, adopted initially or created previously for *Γ_ij_* (Figure 8b).

Subsequently, tetrads of points *S_Aij_*, *S_Bij_*, *S_Cij_* and *S_Dij_* are determined on four side edges *a_ij_*, *b_ij_*, *c_ij_*, *d_ij_* in relation to four adopted vertices *W_ABij_*, *W_CDij_*, *W_ADij_* and *W_BCij_* of *Γ_ij_*, (*i* = 2, *j* = 1) (Figure 8a). The constructed points are the vertices of a quadrangle *S_Aij_S_Bij_S_Cij_S_Dij_* constituting a plane mesh of an auxiliary multi-plane edge net determining a reference surface *ω_r_*. In relation to the reference surface *ω_r_*, four vertices *A_ij_*, *B_ij_*, *C_ij_*, *D_ij_* of the quadrilateral eaves net *B_vij_* are determined. Other tetrads of points *P_Aij_*, *P_Bij_*, *P_Cij_* and *P_Dij_* defining a flat horizontal base of the sought-after free form *∑* are also constructed in relation to four vertices *W_ABij_*, *W_CDij_*, *W_ADij_* and *W_BCij_*. The complex free form *∑* determined by the reference network *Γ* is a sum of all individual free forms *∑_ij_* roofed with *Ω_ij_*.

To construct the subsequent shell sectors *Ω_ij_* of different curvature in two orthogonal directions, the axes *u_ij_* and *v_ij_* must be mutual skew. The greater the curvature diversity of *Ω_ij_* in two orthogonal directions, the greater the distance between the *u_ij_* and *v_ij_* axes must be adopted. To build the diversified shells *Ω_ij_* as sectors of warped surfaces, many pairs of directrices must be modeled with the help of skew straight or curved lines contained in opposite walls of each tetrahedron *Γ_ij_*.

The parameterization proposed in the present article regarding the geometric process of shaping such free forms *∑* is based on an adoption of a finite set of independent variables entering into the proposed novel computer application in the form of division coefficients of the respective pairs of the investigated *Γ*’s vertices. The algorithm of the activities leading to the creation of *Γ* and *∑* is presented in Section 5.

## 5. Results—The Methods Algorithm

**Figure 9:**
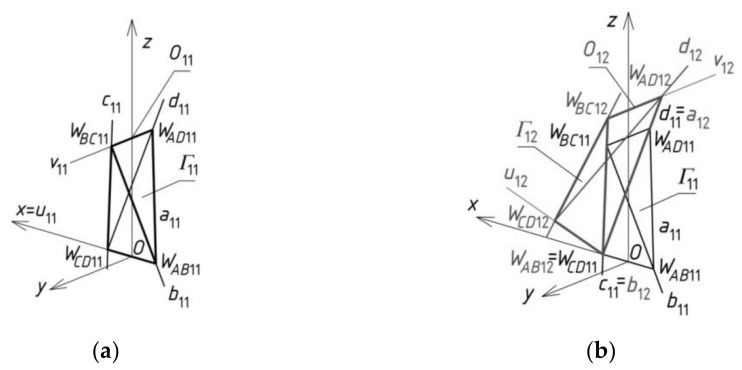
The results of two initial steps of the method’s algorithm used for creating *Γ*: (**a**) *Γ*_11_; (**b**) *Γ*_12_.

The first step of the method’s algorithm relates to the determination of all vertices of the subsequent reference tetrahedrons *Γ_ij_*, constituting the meshes of a reference network *Γ*. The first mesh *Γ*_11_ is created so that the positions of its four vertices *W_AB_*_11_, *W_CD_*_11_, *W_AD_*_11_ and *W_BC_*_11_ are defined. For that purpose, a global coordinate system [*x,y,z*] with the origin *O* was taken (Figure 9a). A first set of the initial data concerning the creation of *Γ*_11_ is formed from the coordinates of these vertices. A few ways of determination of the vertices belonging to the investigated reference networks were developed by Abramczyk [1,17,21]. If we want a sought-after mesh to be symmetric, then its vertices must be arranged either symmetrically, in accordance with the principal planes (*x,z*) and (*y,z*), or in these planes. After defining the vertices *W_AB_*_11_, *W_CD_*_11_, *W_AD_*_11_ and *W_BC_*_11_, we can determine four straight side edges *a*_11_, *b*_11_, *c*_11_ and *d*_11_ of *Γ*_11_.

The second reference tetrahedron *Γ*_12_ is located along the (*x,z*)-plane in one of two principal orthogonal directions relative to *Γ*_11_ (Figure 9b). Its vertex *W_AB_*_12_ is identical to *W_CD_*_11_ introduced previously, so the second set of the initial data is composed of the coordinates of three other vertices *W_CD_*_12_, *W_AD_*_12_ and *W_BC_*_12_ of *Γ*_12_. These vertices can be determined as follows. The positions of the vertices *W_BC_*_12_, *W_AD_*_12_ have to be defined on two side edges *b*_12_ = *c*_11_, *a*_12_ = *d*_11_, by means of two division coefficients of the pairs (*W_CD_*_11_,*W_BC_*_11_) and (*W_CD_*_11_,*W_AD_*_11_) by these vertices. The division coefficients constitute the elements of the second set of the initial data instead of the coordinates of these points.

The position of vertex *W_CD_*_12_ is obtained as a result of a rotation of the triangle *W_AB_*_12_*W_BC_*_12_*W_AD_*_12_ about the axis *v*_12_(*W_AD_*_12_,*W_BC_*_12_) into the position of the triangle *W_CD_*_12_*W_BC_*_12_*W_AD_*_12_ (Figure 9b). If *Γ*_12_ is to be symmetrical towards the (*x,z*)-plane, *W_BC_*_12_ and *W_AD_*_12_ have to be identical to each other with respect to this plane. The point *O*_12_ helpful in programming, is the middle point of *W_AD_*_12_*W_BC_*_12_. Four vertices *W_AB_*_12_, *W_CD_*_12_, *W_AD_*_12_ and *W_BC_*_12_ determine four straight side edges: *a*_12_, *b*_12_, *c*_12_ and *d*_12_ of *Γ*_12_. The remaining tetrahedrons *Γ*_1*j*_ (for *j* > 2) arranged symmetrically along the (*x,z*)-plane are determined in an analogous manner.

**Figure 10:**
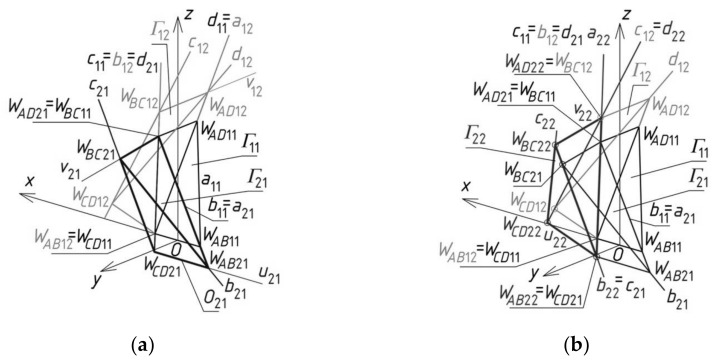
The results of further initial steps of the method’s algorithm used for creating *Γ*: (**a**) *Γ*_21_; (**b**) *Γ*_22_.

The third reference tetrahedron *Γ*_21_ is located in the second of two principal orthogonal directions of *Γ*_11_, that is, along the (*y,z*)-plane (Figure 10a). The third set of the initial data is composed of the coordinates of only three vertices *W_AB_*_21_, *W_CD_*_21_ and *W_BC_*_21_ of *Γ*_21_ because the other vertex *W_AD_*_21_ = *W_BC_*_11_. The positions of the vertices *W_AB_*_21_ and *W_CD_*_21_ are defined on two side edges *a*_21_ = *b*_11_, *d*_21_ = *c*_11_ with the help of two division coefficients belonging to the third set of initial data instead of the coordinates of these points. The position of vertex *W_BC_*_21_ is obtained as a result of a rotation of the triangle *W_AD_*_21_*W_CD_*_21_*W_AB_*_21_ about the axis *u*_21_(*W_CD_*_21_,*W_AB_*_21_) into the position of the triangle *W_BC_*_21_*W_CD_*_21_*W_AB_*_21_. If (*y,z*) is to be the plane of symmetry of *Γ*_21_, then *W_CD_*_21_ and *W_AB_*_21_ have to be isentical to each other in relation to this plane. In addition, the point *O*_21_, helpful in programming, has to be taken as the middle point of *W_CD_*_21_*W_AB_*_21_. Four vertices—*W_AB_*_21_, *W_CD_*_21_, *W_AD_*_21_ and *W_BC_*_21_—determine four straight side edges—*a*_21_, *b*_21_, *c*_21_ and *d*_21_ of *Γ*_21_. The remaining tetrahedrons *Γ_i_*_1_ (for *i* > 2) arranged along the (*y,z*)-plane are determined in an analogous manner.

The fourth reference tetrahedron *Γ*_22_ is located diagonally in relation to *Γ*_11_ (Figure 10b). The fourth set of the initial data should be composed of the coordinates of only two vertices *W_CD_*_22_ and *W_BC_*_22_ because *W_AD_*_22_ = *W_BC_*_12_ and *W_AB_*_22_ = *W_CD_*_21_. The positions of *W_CD_*_22_ and *W_BC_*_22_ are defined on two side edges *d*_22_ = *c*_12_, *b*_22_ = *c*_21_ with the help of two division coefficients belonging to the fourth set of the initial data, instead of the coordinates of these points. Four vertices—*W_AB_*_22_, *W_CD_*_22_, *W_AD_*_22_ and *W_BC_*_22_—determine four straight side edges *a*_22_, *b*_22_, *c*_22_ and *d*_22_ and two axes *u*_22_ and *v*_22_ of *Γ*_22_. The remaining tetrahedrons *Γ_ij_* (for *i,j* > 2) arranged diagonally in relation to *Γ*_11_ are determined in an analogous manner.

Let us follow some selected instructions characteristic of the AutoLISP language of programming the AutoCAD graphic editor used in the novel application to create the investigated reference networks. The procedure presented in Line 1 instructs the displacement of a local coordinate system to the position of the global coordinate system [*x,y,z*]. Line 2 contains the instruction giving the *W_AB_*_12_ point coordinates identical to the coordinates of the point *W_CD_*_11_. Instructions of Lines 3 and 4 calculate the coordinates of the points *W_AD_*_12_ and *W_BC_*_12_ on the side edges *b*_12_ = *c*_11_(*W_CD_*_11_,*W_BC_*_11_) and *a*_12_ = *d*_11_(*W_CD_*_11_,*W_AD_*_11_) by means of two division coefficients—dWBC12 and dWAD12—of the pairs (*W_CD_*_11_,*W_BC_*_11_) and (*W_CD_*_11_,*W_AD_*_11_) by *W_BC_*_12_ and *W_AD_*_12_.

Line 1: (command “luw” “g”)
Line 2: (setq WAB12 (cal “WCD11+Oo”))
Line 3: (setq WBC12 (cal “plt(WCD11,WBC11,dWBC12)”))
Line 4: (setq WAD12 (cal “plt(WCD11,WAD11,dWAD12)”))
Line 5: (setq O12 (cal “plt(WBC12,WAD12,0.5)”))
Line 6: (command “luw” “3” WBC12 WAD12 WAB12)
Line 7: (setq O12u (cal “w2u(O12)”))
Line 8: (command “luw” “_o” O12u)
Line 9: (command “luw” “x” alfCD12)
Line 10: (setq WCD12u (list 0.0 jcd12 0.0) WCD12 (cal “u2w(WCD12u)”))

By means of the instruction from Line 6, a local clockwise coordinate system [*x_L_*,*y_L_*,*z_L_*] with the origin at *W_BC_*_12_ is created so that the positive half axis *x_L_* is determined by the points *W_BC_*_12_ and *W_AD_*_12_, and the positive half axis *y_L_* is contained in the plane (*W_BC_*_12_,*W_AD_*_12_,*W_AB_*_12_), where the point *W_AB_*_12_ determines its positive course. The axis *z_L_* passes through *W_BC_*_12_ perpendicularly to the plane (*x_L_*,*y_L_*), so that, the system [*x_L_*,*y_L_*,*z_L_*] is clockwise. Then, [*x_L_*,*y_L_*,*z_L_*] is moved into a new position [*x_L_*_1_,*y_L_*_1_,*z_L_*_1_] according to the instruction assigned to Line 8. After executing this instruction, the origin of [*x_L_*_1_,*y_L_*_1_,*z_L_*_1_] is located in the middle point *O*_12_ of *W_BC_*_12_*W_AD_*_12_ (Figure 9b).

Since the translation of the old system [*x_L_*,*y_L_*,*z_L_*] to its new position [*x_L_*_1_,*y_L_*_1_,*z_L_*_1_] has to be performed, the coordinates of the point *O*_12_ must be transformed into the new system. This action is initiated by the procedure shown in Line 7. The instruction given in Line 9 rotates the system [*x_L_*_1_,*y_L_*_1_,*z_L_*_1_] to its new position [*x_L_*_2_,*y_L_*_2_,*z_L_*_2_]. The point *W_CD_*_12_*_u_* takes the coordinates (0.0 jcd12 0.0) in its new position, according to the instruction given in Line 10. The height of the triangle *W_AB_*_12_*W_BC_*_12_*W_AD_*_12_, passed from the point *W_AB_*_12_ is equal to jcd12. In the second part of Line 10, the coordinates of *W_CD_*_12_ are transformed from the local system [*x_L_*_2_,*y_L_*_2_,*z_L_*_2_] to the global system [*x,y,z*].

The code of the presented application contains two main instructions (while...) for creating subsequent meshes *Γ_ij_* located in two orthogonal directions, that is, in *i* rows and *j* columns of the network *Γ*. The selection function (cond...) makes it possible to distinguish and determine four sets of these meshes. The first set relates to the first mesh *Γ*_11_. The subsequent two sets concern the meshes located in two orthogonal directions passing along the planes (*x,z*) and (*y,z*). The last set of meshes is related to the diagonal directions relative to *Γ*_11_.


(setq i 0)

(while (<= i iN)

(setq j 0)

(setq i (+ i 1))

(while (<= j jN)

(setq j (+ j 1))

  (cond ((and (= i 1) (= j 1))  (progn … creation of the first *Γ*_11_))

    ((and (= i 1) (> j 1))  (progn … creation of the first type of orthogonal reference tetrahedrons *Γ*_1*j*_))

    ((and (> i 1) (= j 1))  (prong … creation of the second type of orthogonal reference tetrahedrons *Γ_i_*_1_))

    ((and (> i 1) (> j 1))  (progn … creation of diagonal reference tetrahedrons *Γ_ij_*)

  );cond

);while.

);while


**Figure 11:**
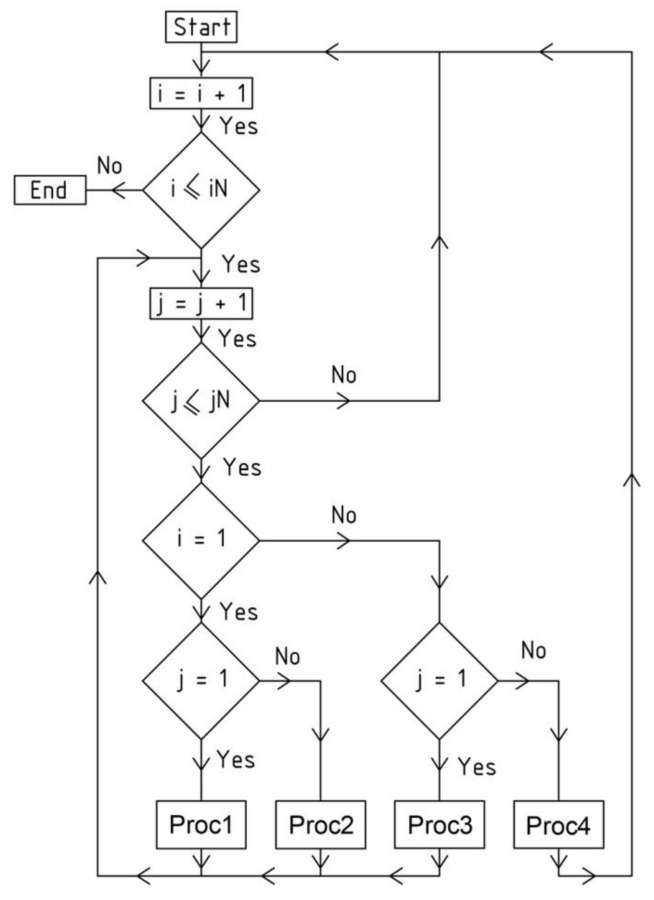
The algorithm of the iterative process for creating the nets *Γ* and *B_v_* and structures *Ω* and *∑*.

The above-mentioned procedures are represented by the block scheme shown in Figure 11. They are the main part of the method’s algorithm presented in this article.

**Figure 12:**
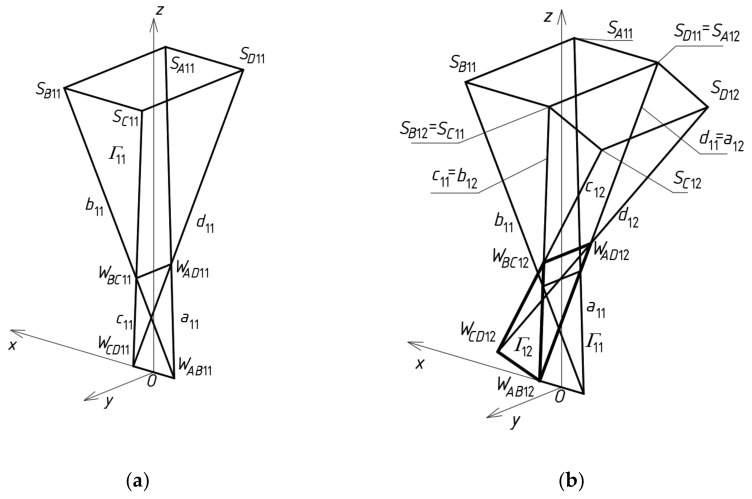
Tetrads of the *ω_r_*‘s points belonging to: (**a**) *Γ*_11_; (**b**) *Γ*_12_.

To determine four points *S_A_*_11_, *S_B_*_11_, *S_C_*_11_ and *S_D_*_11_ (Figure 12a) defining a sector of a reference surface, four division coefficients constituting four elements of the first set of the initial data must be employed. Subsequently, four auxiliary points of the reference surface must be determined on the side edges of *Γ*_12_ so that two of them *S_B_*_12_ = *S_C_*_11_, *S_A_*_12_ = *S_D_*_11_ belong to *b*_12_ = *c*_11_ and *a*_12_ = *d*_11_, and the other two—*S_C_*_12_ and *S_D_*_12_—are determined on two side edges *c*_12_ and *d*_12_ using two division coefficients constituting two elements of the second set of the initial data adopted earlier (Figure 12b). Tetrads of points *S_A_*_1*j*_, *S_B_*_1*j*_, *S_C_*_1*j*_ and *S_D_*_1*j*_ belonging to the remaining tetrahedrons *Γ*_1*j*_ (for *j* > 2) arranged along the (*x,z*)-plane are determined in an analogous manner.

**Figure 13:**
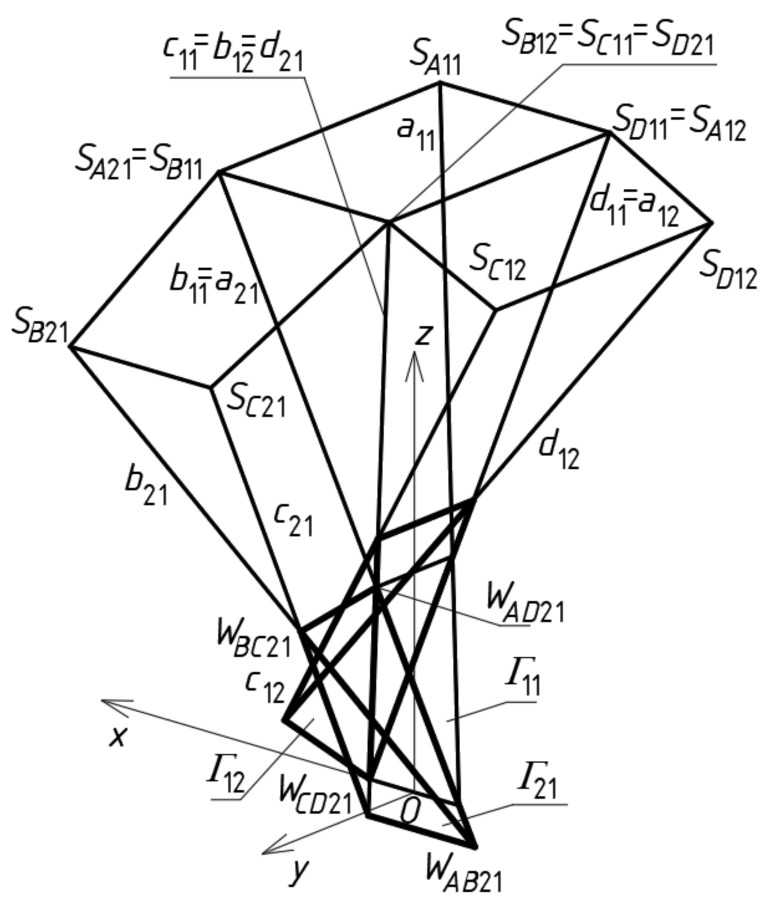
Subsequent tetrads of the points belonging to *Γ*_21_.

At the subsequent step of the method’s algorithm, four auxiliary points *S_A_*_21_, *S_B_*_21_, *S_C_*_21_ and *S_D_*_21_ of the reference surface must be determined on the side edges of *Γ*_21_, so that two of them are *S_A_*_21_ = *S_B_*_11_, *S_D_*_21_ = *S_C_*_11_, and the other two *S_C_*_21_ and *S_B_*_21_ are determined on two side edges *c*_21_ and *b*_21_ (Figure 13), using two division coefficients of the third set of the initial data.

**Figure 14:**
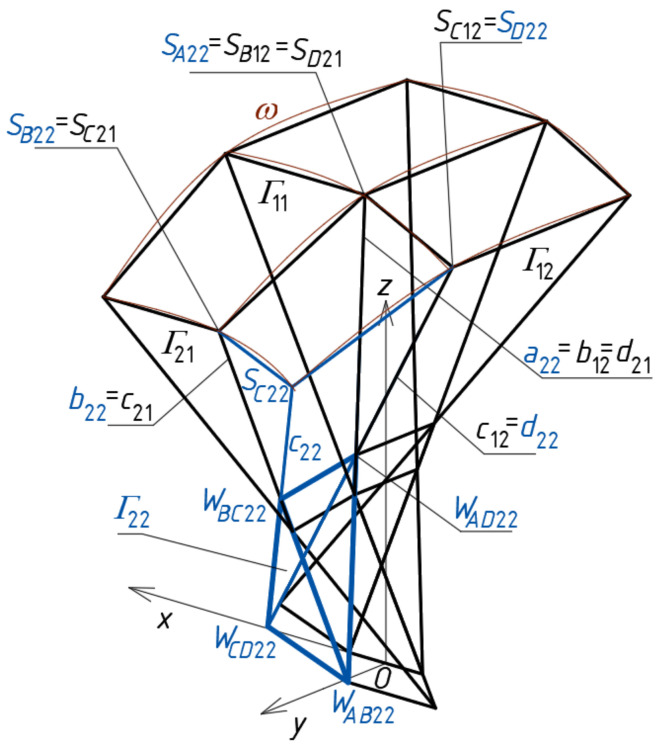
An auxiliary quadrilateral net determining a reference surface *ω* the polyhedral net *Γ*.

At the end of this step, four auxiliary points f the *ω_r_* reference surface are determined on the side edges of *Γ*_22_. Three of these points are determined on *a*_22_ = *b*_12_, *b*_22_ = *c*_21_, and *d*_22_ = *c*_12_, so that *S_A_*_22_ = *S_B_*_12_, *S_B_*_22_ = *S_C_*_21_ and *S_D_*_22_ = *S_C_*_12_ (Figure 14). The last one belongs to *c*_22_ determined by *W_CD_*_22_ and *W_BC_*_22_, using a division coefficient constituting one element of the fourth set of the initial data.

Tetrads of points *S_Aij_*, *S_Bij_*, *S_Cij_* and *S_Dij_* belonging to the remaining tetrahedrons *Γ_ij_* (for *i,j* > 2) arranged diagonally in relation to *Γ*_11_ have to be determined in an analogous manner. On the basis of the auxiliary quadrilateral net having vertices located at the points *S_Aij_*, *S_Bij_*, *S_Cij_* and *S_Dij_* of *Γ_ij_*, the *ω_r_* surface is defined (Figure 14).

Some procedures related to the determination of the aforementioned points *S_A__ij_*, *S_B__ij_*, *S_C__ij_* and *S_D__ij_* are presented and analyzed below. The procedures shown in Lines 11 to 14 realize the calculations of the coordinates of the above points. The procedure given in Line 15 creates the plane quadrangle *S_A_*_11_*S_B_*_11_*S_C_*_11_*S_D_*_11_ in the three-dimensional computer space.

Line 11: (setq SA11 (cal “plt(WAB11,WAD11,dSA11)”))
Line 12: (setq SB11 (cal “plt(WAB11,WBC11,dSB11)”))
Line 13: (setq SD11 (cal “plt(WCD11,WAD11,dSD11)”))
Line 14: (setq SC11 (cal “plt(WCD11,WBC11,dSC11)”))
Line 15: (command “linia” SA11 SB11 SC11 SD11 SA11 ““)

The instructions assigning the values of the coordinates of the previously determined points *S_D_*_11_ and *S_C_*_11_ to the coordinates of *S_A_*_12_ and *S_B_*_12_ are presented in Lines 16 and 17. The procedure from Line 18 calculates the division coefficient of the pair (*W_AB_*_12_,*W_AD_*_12_) by *S_A_*_12_ using an internal novel function (wspolcz...) written by the author. The next instruction located in Line 19 assigns the value calculated by the function (wspolcz...) to the division coefficient dSA12.

The analogous procedures resulting in the calculation of the coefficient dSB12 of the pair (*W_AB_*_12_,*W_BC_*_12_) by *S_B_*_12_ are given in Lines 20 and 21. The instructions from Lines 22 and 23 calculate the coordinates of the points *S_C_*_12_ and *S_D_*_12_ positioned on the straight lines (*W_CD_*_12_,*W_BC_*_12_) and (*W_CD_*_12_,*W_AD_*_12_), using the division coefficients dWBC12 and dWAD12 of the aforementioned pairs by *S_C_*_12_ and *S_D_*_12_. The procedure shown in Line 24 creates a quadrangle *S_A_*_12_*S_B_*_12_*S_C_*_12_ *S_D_*_12_ in the three-dimensional computer space.

Line 16: (setq SA12 (cal “SD11 + Oo”))
Line 17: (setq SB12 (cal “SC11 + Oo”))
Line 18: (wspolcz SA12 WAB12 WAD12)
Line 19: (setq dSA12 wspcz)
Line 20: (wspolcz SB12 WAB12 WBC12)
Line 21: (setq dSB12 wspcz)
Line 22: (setq SC12 (cal “plt(WCD12,WBC12,dSC12)”))
Line 23: (setq SD12 (cal “plt(WCD12,WAD12,dSD12)”))
Line 24: (command “linia” SA12 SB12 SC12 SD12 SA12 ““)

The procedures calculating the coordinates of *S_A_*_22_, *S_B_*_22_, *S_C_*_22_ and *S_D_*_22_ are analogous to those presented earlier for *Γ*_11_ and *Γ*_12_. The internal function (wspolcz...) must be used three times to calculate the coordinates of the above three points. In the case of *Γ*_22_, the value of the calculated division coefficient dSA22 of (*W_AB_*_22_,*W_AD_*_22_) by *S_C_*_11_ have to be assigned to the division coefficient dSC22 of (*W_CD_*_22_,*W_BC_*_22_) by *S_C_*_2_.

It is worth paying attention to the following properties of the reference network *Γ* built so far. All vertices of each reference tetrahedron *Γ_ij_* designate four side edges *a_ij_*, *b_ij_*, *c_ij_* and *d_ij_* and four planes of *Γ*. Each new reference tetrahedron *Γ_i_*_+1*j*_ or *Γ_ij_*_+1_ is created as a spatial mesh with two sought-after vertices defined on two side edges of two previously constructed tetrahedrons *Γ_ij_*, so the subsequent pairs of adjacent meshes of *Γ* have to have common planes. The roof directrices of each *Γ_ij_* should be positioned in these planes.

**Figure 15:**
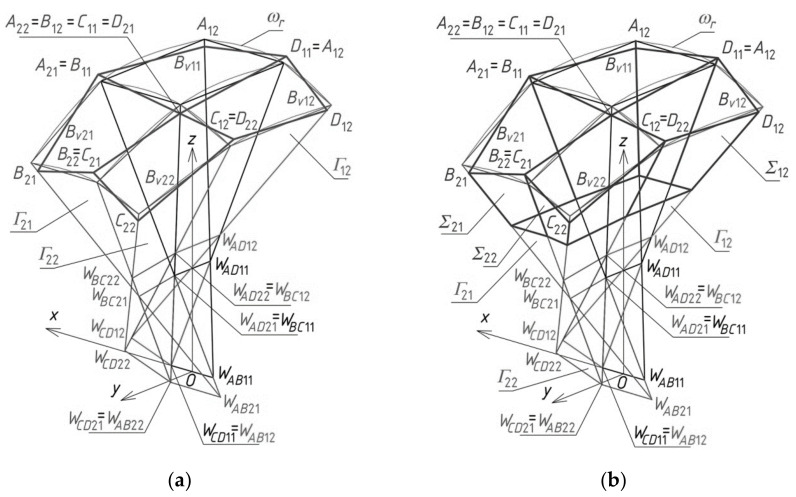
Final steps of creating: (**a**) *B_v_* net; (**b**) elevation structure *∑*.

To calculate the coordinates of *A*_11_, *B*_11_, *C*_11_ and *D*_11_ of a closed spatial eaves quadrangle *B_v_*_11_, four division coefficients constituting four elements of the first set of the initial data, used for *Γ*_11_, have to be adopted. These points belong to four side edges *a*_11_(*W_AB_*_11_,*W_AD_*_11_), *b*_11_(*W_AB_*_11_,*W_BC_*_11_), *c*_11_(*W_BC_*_11_,*W_CD_*_11_) and *d*_11_(*W_CD_*_11_,*W_AD_*_11_) and should be positioned in accordance with *ω_r_* by means of the respective division coefficients (Figure 15a). Subsequently, four vertices—*A*_12_, *B*_12_, *C*_12_ and *D*_12_ of *B_v_*_12_—have to be determined on the side edges *a*_12_, *b*_12_, *c*_12_ and *d*_12_ of *Γ*_12_ in relation to *ω_r_*. Two of these vertices are *A*_12_ = *C*_11_, *B*_12_ = *D*_11_. The other two *C*_12_ and *D*_12_ have to be determined on two side edges *c*_12_(*W_BC_*_12_,*W_CD_*_12_) and *d*_12_(*W_CD_*_12_,*W_AD_*_12_) using two division coefficients constituting two elements of the second set of the initial data. The tetrads of points *A*_1*j*_, *B*_1*j*_, *C*_1*j*_ and *D*_1*j*_ belonging to the side edges of the remaining tetrahedrons *Γ*_1*j*_ (for *j* > 2) and constituting the vertices of the remaining spatial quadrangles *B_v_*_1*j*_ located along the (*x,z*)-plane are determined in an analogous manner.

Four vertices *A*_21_, *B*_21_, *C*_21_ and *D*_21_ of the eaves *B_v_*_21_ have to be determined on the side edges *a*_21_, *b*_21_, *c*_21_ and *d*_21_ of *Γ*_21_ in accordance with *ω_r_* (Figure 15a). Two of these vertices are *A*_21_ = *B*_11_, *D*_21_ = *C*_11_. The other two *C*_21_ and *B*_21_ have to be determined on two side edges *c*_21_ (*W_BC_*_21_,*W_CD_*_21_) and *b*_21_(*W_AB_*_21_,*W_BC_*_21_), using two division coefficients constituting two elements of the third set of the initial data. The points *A_i_*_1_, *B_i_*_1_, *C_i_*_1_ and *D_i_*_1_ belonging to the other tetrahedrons *Γ_i_*_1_ and constituting the vertices of the spatial quadrangles *B_vi_*_1_ (for *i* > 2) arranged along the (*y,z*)-plane are determined in an analogous manner.

Four vertices *A*_22_, *B*_22_, *C*_22_ and *D*_22_ of the quadrangle *B_v_*_22_ have to be determined on the side edges *a*_22_(*W_AB_*_22_,*W_AD_*_22_), *b*_22_(*W_AB_*_22_,*W_BC_*_22_), *c*_22_(*W_BC_*_22_,*W_CD_*_22_) and *d*_22_(*W_CD_*_22_,*W_AD_*_22_) of *Γ*_22_ relative to *ω_r_* (Figure 15a). Three of these points are determined on *a*_22_ = *c*_11_, *b*_22_ = *c*_21_, and *d*_22_ = *c*_12_ so that *A*_22_ = *C*_11_, *B*_22_ = *C*_21_ and *D*_22_ = *C*_12_. The last one *C*_22_ belongs to *c*_22_(*W_CD_*_22_,*W_BC_*_22_). It is determined by means of one division coefficient constituting an element of the fourth set of the initial data. The tetrads of points *A_ij_*, *B_ij_*, *C_ij_* and *D_ij_* belonging to the remaining tetrahedrons *Γ_ij_* (for *i,j* > 2) arranged diagonally with regard to *Γ*_22_ can be determined in an analogous manner.

In order to determine a horizontal plane base of the free form *∑*, one its point, for example, *P_D_*_12_ has to be defined on *d*_12_ (Figure 15b). The value of the division coefficient of the pair (*W_CD_*_12_,*W_AD_*_12_) by this point is an element of the second set of the initial data. Another point of the plane base is the intersection of the horizontal base plane passing through *P_D_*_12_ with the subsequent tetrads of side edges of *Γ*_11_, *Γ*_12_, etc.

The result of adding up the four reference tetrahedrons *Γ_ij_* is a subnet *Γ*_1_ constituting a quarter of the resultant reference network *Γ*. The other three parts of *Γ* can be built using a *z*-axis symmetry and two (*x,z*)-plane and (*y,z*)-plane symmetries, called 3D-mirrors, in the way described in the next section on an example of a more complex reference network.

Similarly, four tetrahedrons *∑_ij_* create a subnet *∑*_1_ constituting one-fourth of the designed building free form *∑*. Four eaves quadrangles *B_vij_* create a subnet *B_v_*_1_ constituting one-fourth of the network *B_v_*. On the basis of *B_v_*, the roof structure *Ω* composed of many sectors *Ω_ij_* is created.

In summary, for the case of creating reference tetrahedrons *Γ*_1*j*_ or *Γ_i_*_1_ (for *j* > 2 or *i* > 2 and *i, j* different from (1) located in two orthogonal directions of planes (*x,z*) and (*y,z*), one vertex of each of these tetrahedrons must be located outside the side edges of the already created subnet of *Γ*. The vertex determines a new plane of *Γ* passing through the already constructed axis of the designed tetrahedron. Two of its subsequent vertices ought to be determined on two side edges of the previously created subnet of *Γ* with the help of two division coefficients. The location of the fourth vertex is identical with one of the vertices of *Γ* constructed earlier. In the case of the diagonal directions of *Γ*_11_, each new reference tetrahedron *Γ_ij_* has two vertices identical to two previously constructed vertices of *Γ*. However, its new vertices have to be determined on two side edges of the previously created subnet of *Γ* by means of the respective division coefficients.

This way of constructing the subsequent reference tetrahedrons located in the orthogonal and diagonal courses of each reference network *Γ* is characterized by the fact that each inner side edge of *Γ* is shared by four adjacent reference tetrahedrons and eight vertices of these four tetrahedrons belong to the same side edge of *Γ*. In a general case, these eight vertices occupy four different positions, in pairs. This topic is going to be presented in further publications. The example of using the proposed algorithm for determining the parametric reference polyhedral networks *Γ* and eaves nets *B_v_* is given in next section.

## 6. Results—Parametric Shaping of the Reference Networks

Two ways of shaping of the investigated networks *Γ* and *B_v_* can be carried out in scientific and engineering problems. One of these ways is based on the stiff-motions such as translations and rotations of several initially adopted or calculated points edges and planes determining the other vertices and side edges of these nets [22]. The second way, presented in this article, is more intuitive, because it enables one to create parametric models by means of the division coefficients expressing very specific relations between the main elements of the designed building free-forms. To make this method easy for many designers, a novel computer application written in the AutoLISP language of programming the AutoCAD visual editor was developed.

The last way requires a little more operations related to the division coefficients of the respective pairs of the determined vertices of the investigated reference polyhedral *Γ* and the quadrilateral *B_v_* networks. The coefficients define the positions of: (1) the vertices of the sought-after reference network *Γ* with respect to a few intuitively adopted specific points of *Γ*, (2) the planes of *Γ*, (3) the points *S_Aij_*, *S_Bij_*, *S_Cij_*, and *S_Dij_* belonging to *ω_r_*, and (4) the vertices *A_ij_ B_ij_*, *C_ij_* and *D_ij_* of *B_v_* determining the multi-shell roof structure *Ω*.

**Figure 16:**
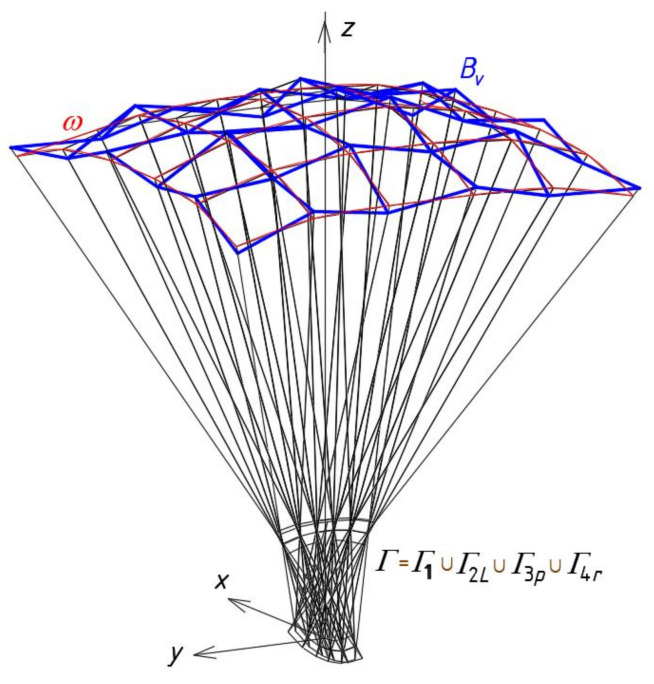
A multi-segment reference network *Γ* consisting of four symmetric subnets *Γ*_1_, *Γ*_2*L*_, *Γ*_3*p*_ and *Γ*_4*r*_.

In the example presented below, a usage of the method for computational determining one quarter *Γ*_1_ of a reference network *Γ* (Figure 16) based on some adopted proportions is discussed. All vertices of three other quarters *Γ*_2*L*_, *Γ*_3*p*_, *Γ*_4*r*_ of *Γ* are determined using: (1) *z*-axial symmetry for the case of *Γ*_2*L*_, (2) (*x,z*)-plane symmetry called 3D-mirror for *Γ*_3*p*_, and (3) (*y,z*)-plane symmetry for the case of *Γ*_4*r*_. A description of creating the symmetric nets *Γ*_2*L*_, *Γ*_3*p*_, *Γ*_4*r*_ is not presented in this article.

**Figure 17:**
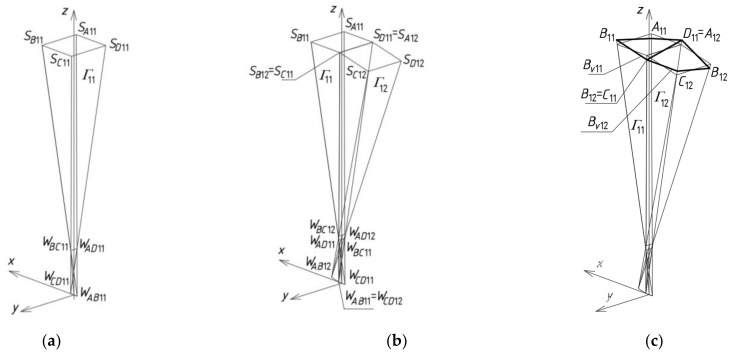
Two initial steps of the method’s algorithm related to creation of: (**a**) *Γ*_11_; (**b**) *Γ*_12_; (**c**) *B_v_*_11_ and *B_v_*_12_.

Creating one quarter *Γ*_1_ of an *z*-axially symmetric network *Γ* is started by defining the first *z*-axis-symmetric mesh *Γ*_11_ (Figure 17a). It is continued for subsequent meshes *Γ_ij_* arranged orthogonally (Figure 17b) and, next, diagonally with respect to *Γ*_11_, following the algorithm presented in the previous section. To obtain the first tetrahedron *Γ*_11_, four of its vertices—*W_AB_*_11_, *W_CD_*_11_, *W_AD_*_11_ and *W_BC_*_11_—are defined by means of their coordinates listed in Table 1.

The positions of the points *S_A_*_11_, *S_B_*_11_, *S_C_*_11_ and *S_D_*_11_ of *ω_r_* are defined with the following division coefficients (*W_AB_*_11_,*W_AD_*_11_)\*S_A_*_11_, (*W_AB_*_11_,*W_BC_*_11_)\*S_B_*_11_, (*W_CD_*_11_,*W_BC_*_11_)\*S_C_*_11_ and (*W_CD_*_11_,*W_AD_*_11_)\*S_D_*_11_, where

(*W_AB_*_11_,*W_AD_*_11_)\*S_A_*_11_ = $m(\vec{W_{AB11}S_{A11}})$/$m(\vec{W_{AB11}W_{AD11}})$

(*W_AB_*_11_,*W_BC_*_11_)\*S_B_*_11_ = $m(\vec{W_{AB11}S_{B11}})$/$m(\vec{W_{AB11}W_{BC11}})$

(*W_CD_*_11_,*W_BC_*_11_)\*S_C_*_11_ = $m(\vec{W_{CD11}S_{C11}})$/$m(\vec{W_{CD11}W_{BC11}})$

(*W_CD_*_11_,*W_AD_*_11_)\*S_D_*_11_ = $m(\vec{W_{CD11}S_{D11}})$/$m(\vec{W_{CD11}W_{AD11}})$

and $\vec{W_{AB11}W_{AD11}}$ is the vector starting with *W_AB_*_11_ and ending at *W_AD_*_11_, m($\vec{W_{AB11}W_{AD11}}$) is the measure of $\vec{W_{AB11}W_{AD11}}$, $\vec{W_{AB11}S_{A11}}$ is the vector with the starting point at *W_AB_*_11_ and the ending point at *S_A_*_11_, etc. The values of the above ratios are listed in Table 2. The subsequent points *S_A_*_11_*, S_B_*_11_, *S_C_*_11_ and *S_D_*_11_ define the spatial quadrangle determining a respective segment of *ω_r_*.

The locations of the vertices *A*_11_, *B*_11_, *C*_11_ and *D*_11_ of *Γ*_11_ (Figure 17c) are defined by means of the aforementioned vertices of *Γ*_11_ and the following proportions

(*W_AB_*_11_,*W_AD_*_11_)\*A*_11_ = $m(\vec{W_{AB11}A_{11}})$/$m(\vec{W_{AB11}W_{AD11}})$

(*W_AB_*_11_, *W_BC_*_11_)\*B*_11_ = $m(\vec{W_{AB11}B_{11}})$/$m(\vec{W_{AB11}W_{BC11}})$

(*W_CD_*_11_, *W_BC_*_11_)\*C*_11_ = $m(\vec{W_{CD11}C_{11}})$/$m(\vec{W_{CD11}W_{BC11}})$

(*W_CD_*_11_, *W_AD_*_11_)\*D*_11_ = $m(\vec{W_{CD11}D_{11}})$/$m(\vec{W_{CD11}W_{AD11}})$

where $\vec{W_{AB11}A_{11}}$ is the vector defined by the starting point at *W_AB_*_11_ and the ending point *A*_11_, etc. The points *A*_11_, *B*_11_, *C*_11_ and *D*_11_ determine the spatial quadrangle *B_v_*_11_ limiting the single smooth shell segment *Ω*_11_ of the complex roof structure *Ω*. Subsequently, the values of four division coefficients (*W_AB_*_11_,*W_AD_*_11_)\(*S_A_*_11_,*A*_11_), (*W_AB_*_11_,*W_BC_*_11_)\(*S_B_*_11_,*B*_11_), (*W_CD_*_11_,*W_BC_*_11_)\(*S_C_*_11_,*C*_11_) and (*W_CD_*_11_,*W_AD_*_11_)\(*S_D_*_11_,*D*_11_) must be adopted as follows

(*W_AB_*_11_,*W_AD_*_11_)\(*S_A_*_11_,*A*_11_) = $m(\vec{S_{A11}A_{11}})$/$m(\vec{W_{AB11}W_{AD11}})$ = (*W_AB_*_11_,*W_AD_*_11_)\*A*_11_-(*W_AB_*_11_,*W_AD_*_11_)\*S_A_*_11_

(*W_AB_*_11_,*W_BC_*_11_)\(*S_B_*_11_,*B*_11_) = $m(\vec{S_{B11}B_{11}})$/$m(\vec{W_{AB11}W_{BC11}})$ = (*W_AB_*_11_,*W_BC_*_11_)\*B*_11_–(*W_AB_*_11_,*W_BC_*_11_)\*S_B_*_11_

(*W_CD_*_11_,*W_BC_*_11_)\(*S_C_*_11_,*C*_11_) = $m(\vec{S_{C11}C_{11}})$/$m(\vec{W_{CD11}W_{BC11}})$ = (*W_CD_*_11_,*W_BC_*_11_)\*C*_11-_(*W_CD_*_11_,*W_BC_*_11_)\*S_C_*_11_

(*W_CD_*_11_,*W_AD_*_11_)\(*S_D_*_11_,*D*_11_) = $m(\vec{S_{D11}D_{11}})$/$m(\vec{W_{CD11}W_{AD11}})$ = (*W_CD_*_11_,*W_AD_*_11_)\*D*_11-_(*W_CD_*_11_,*W_AD_*_11_)\*S_D_*_11_

The above constants have positive or negative signs depending on whether the points *A*_11_, *B*_11_, *C*_11_ and *D*_11_ lie above or below *ω_r_* defined by means of the quadrangle *S_A_*_11_*S_B_*_11_*S_C_*_11_*S_D_*_11_ (Figure 17a).

If we transform the above formulas, the division ratios (*W_AB_*_11_,*W_AD_*_11_)\*A*_11_, (*W_AB_*_11_,*W_BC_*_11_)\*B*_11_, (*W_CD_*_11_,*W_BC_*_11_)\*C*_11_ and (*W_CD_*_11_,*W_AD_*_11_)\*D*_11_ can be calculated as follows

(*W_AB_*_11_,*W_AD_*_11_)\*A*_11_ = (*W_AB_*_11_,*W_AD_*_11_)\*S_A_*_11_ + (*W_AB_*_11_,*W_AD_*_11_)\(*S_A_*_11_,*A*_11_)

(*W_AB_*_11_,*W_BC_*_11_)\*B*_11_ = (*W_AB_*_11_,*W_BC_*_11_)\*S_B_*_11_ + (*W_AB_*_11_,*W_BC_*_11_)\(*S_B_*_11_,*B*_11_)

(*W_CD_*_11_,*W_BC_*_11_)\*C*_11_ = (*W_CD_*_11_,*W_BC_*_11_)\*S_C_*_11_ + (*W_CD_*_11_,*W_BC_*_11_)\(*S_C_*_11_,*C*_11_)

(*W_CD_*_11_,*W_AD_*_11_)\*D*_11_ = (*W_CD_*_11_,*W_AD_*_11_)\*S_D_*_11_ + (*W_CD_*_11_,*W_AD_*_11_)\(*S_D_*_11_,*D*_11_)

The values of the division coefficients used in the example are given in Table 2.

In order to create the tetrahedron *Γ*_12_, four of its vertices—*W_AB_*_12_, *W_CD_*_12_, *W_AD_*_12_ and *W_BC_*_12_—were defined by means of the coordinates listed in Table 3.

The positions of the points *S_A_*_12_*, S_B_*_12_, *A*_12_ and *B*_12_ are similar to the positions of *S_D_*_11_, *S_C_*_11_, *D*_11_ and *C*_11_, so the following division coefficients have to be calculated as follows

(*W_AB_*_12_,*W_AD_*_12_)\*S_A_*_12_ = (*W_CD_*_11_,*W_AD_*_11_)\*S_D_*_11_/(*W_CD_*_11_,*W_AD_*_11_)\*W_AD_*_12_

(*W_AB_*_12_,*W_BC_*_12_)\*S_B_*_12_ = (*W_CD_*_11_,*W_BC_*_11_)\*S_C_*_11_/(*W_CD_*_11_,*W_BC_*_11_)\*W_BC_*_12_

(*W_AB_*_12_,*W_AD_*_12_)\*A*_12_ = (*W_CD_*_11_,*W_AD_*_11_)\*D*_11_/(*W_CD_*_11_,*W_AD_*_11_)\*W_AD_*_12_

(*W_AB_*_12_,*W_BC_*_12_)\*B*_12_ = (*W_CD_*_11_,*W_BC_*_11_)\*C*_11_/(*W_CD_*_11_,*W_BC_*_11_)\*W_BC_*_12_

where the spaces before and behind the slash denote that we have common division of two numbers. Other division ratios related to *Γ*_12_ and *B_v_*_12_ are adopted as follows.

(WCD12,WBC12)\SC12 = (WAB12,WAD12)\SA12

(WCD12,WAD12)\SD12 = (WAB12,WBC12)\SB12

(WCD12,WBC12)\C12 = (WAB12,WAD12)\A12

(WCD12,WAD12)\D12 = (WAB12,WBC12)\B12

The calculated values of these division coefficients are given in Table 4.

The positions of the points: (1) *S_A_*_13_, *S_B_*_13_, *S_C_*_13_, *S_D_*_13_, *A*_13_, *B*_13_, *C*_13_ and *D*_13_, (2) *S_A_*_21_, *S_B_*_21_, *S_C_*_21_, *S_D_*_21_, *A*_21_, *B*_21_, *C*_21_ and *D*_21_, (3) *S_A_*_31_, *S_B_*_31_, *S_C_*_31_, *S_D_*_31_, *A*_31_, *B*_31_, *C*_31_ and *D*_31_ can be defined or calculated in an analogous way, as for *S_A_*_12_, *S_B_*_12_, *S_C_*_12_, *S_D_*_12_, *A*_12_, *B*_12_, *C*_12_ and *D*_12_. The values of the coordinates of all aforementioned points are given in Table A2 and Table A3, posted in Appendix A.

**Figure 18:**
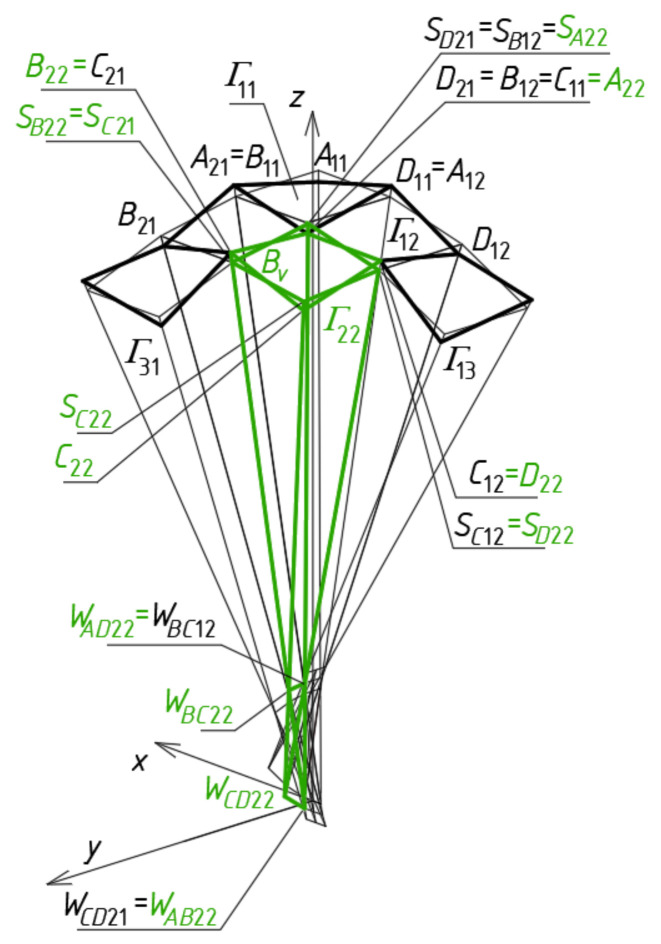
The reference tetrahedron *Γ*_22_ and spatial quadrangle *B_v_*_22_.

To determine the last tetrahedron *Γ*_22_ investigated in our example, four of its vertices—*W_AB_*_22_, *W_CD_*_22_, *W_AD_*_22_ and *W_BC_*_22_ (Figure 18)—are initially defined by means of the coordinates listed in Table 5.

To construct the quadrangle *B_v_*_22_ (Figure 18), the values of the vertices *S_A_*_22_*, S_B_*_22_, *S_C_*_22_, *S_D_*_22_, *A*_22_, *B*_22_, *C*_22_ and *D*_22_ must be calculated in the following way. The positions of the points *S_A_*_22_*, S_B_*_22_, *S_D_*_22_, *A*_22_, *B*_22_ and *D*_22_ are similar to the positions of *S_C_*_11_, *S_C_*_21_, *S_C_*_12_, *C*_11_, *C*_21_ and *C*_12_ determined previously for *Γ*_11_, *Γ*_21_, *Γ*_12_, *B_v_*_11_, *B_v_*_21_ and *B_v_*_12_.

To achieve the coordinates of the aforementioned points of *ω_r_* and *B_v_*, the following division coefficients should be calculated

(*W_AB_*_22_,*W_AD_*_22_)\*S_A_*_22_ = $\frac{(WCD11,WBC11)\backslash SC11}{(WCD11,WBC11)\backslash WAD22\cdot (WBC11,WCD11)\backslash WAB22}$

(*W_AB_*_22_,*W_AD_*_22_)\*A*_22_ = $\frac{(WCD11,WBC11)\backslash C11}{(WCD11,WBC11)\backslash WAD22\cdot (WBC11,WCD11)\backslash WAB22}$

(*W_AB_*_22_,*W_AD_*_22_)\(*S_A_*_22_,*A*_22_) = (*W_AB_*_22_,*W_AD_*_22_)\*A*_22_ − (*W_AB_*_22_,*W_AD_*_22_)\*S_A_*_22_

(*W_AB_*_22_,*W_BC_*_22_)\*S_B_*_22_ = (*W_CD_*_21_,*W_BC_*_21_)\*S_C_*_21_/(*W_CD_*_21_,*W_BC_*_21_)\*W_BC_*_22_

(*W_AB_*_22_,*W_BC_*_22_)\*B*_22_ = (*W_CD_*_21_,*W_BC_*_21_)\*C*_21_/(*W_CD_*_21_,*W_BC_*_21_)\*W_BC_*_22_

(*W_AB_*_22_,*W_BC_*_22_)\(*S_B_*_22_,*B*_22_) = (*W_AB_*_22_,*W_BC_*_22_)\*B*_22_ − (*W_AB_*_22_,*W_BC_*_22_)\*S_B_*_22_

(*W_CD_*_22_,*W_AD_*_22_)\*S_D_*_22_ = (*W_CD_*_12_,*W_AD_*_12_)\*S_C_*_12_/(*W_BC_*_12_,*W_CD_*_12_)\*W_CD_*_22_

(*W_CD_*_22_,*W_AD_*_22_)\*D*_22_ = (*W_CD_*_12_,*W_AD_*_12_)\*C*_12_/(*W_BC_*_12_,*W_CD_*_12_)\*W_CD_*_22_

(*W_CD_*_22_,*W_AD_*_22_)\(*S_D_*_22_,*D*_22_) = (*W_CD_*_22_,*W_AD_*_22_)\*D*_22_ − (*W_CD_*_22_,*W_AD_*_22_)\*S_D_*_22_

A detailed description of a mutual position of the vertices belonging to one exemplary configuration of a polygonal *B_v_* net and a polyhedral net *Γ* is presented by Abramczyk using the method based on stiff motions [22]. In the present article, the extensive method of parametric shaping of the regular roof shell structures by means of the orthotropic properties of the corrugated shell sectors is developed based on the division coefficients.

The spaces before and after slash/depict if a usual division is used. In contrast, a division ratio is expressed if there are not spaces before and after the backslash\. On the basis of the above division coefficients, the following can be calculated

(*W_CD_*_22_,*W_BC_*_22_)\*S_C_*_22_ = (*W_AB_*_22_,*W_AD_*_22_)\*S_A_*_22_

(*W_CD_*_22_,*W_BC_*_22_)\*C*_22_ = (*W_AB_*_22_,*W_AD_*_22_)\*A*_22_

(*W_CD_*_22_,*W_BC_*_22_)\(*S_C_*_22_,*C*_22_) = (*W_CD_*_22_,*W_BC_*_22_)\*C*_22_−(*W_CD_*_22_,*W_BC_*_22_)\*S_C_*_22_

The values of these division coefficients are given in Table 6.

**Figure 19:**
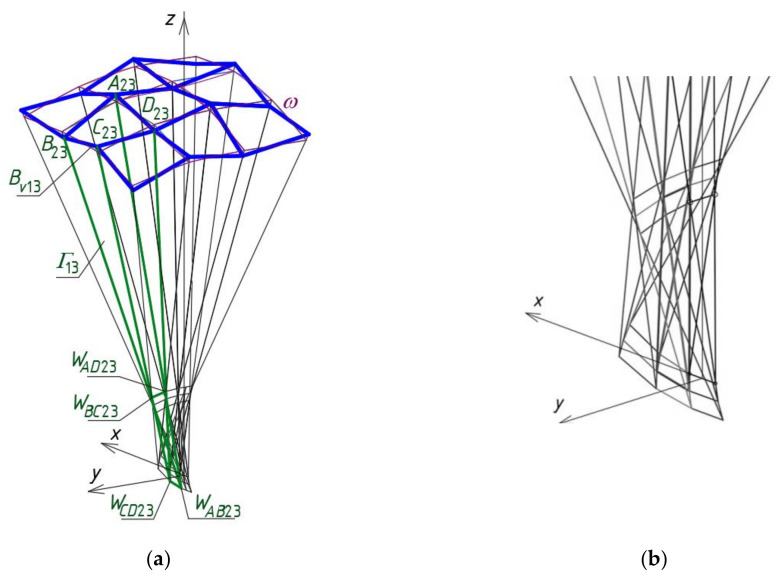
(**a**) The first quarter of the reference network *Γ* composed of *Γ_ij_* for *i,j* = 1 to 3, (**b**) the *Γ*’s vertices.

Analogous proportions as for the quadrangle *Γ*_22_ can be defined for other meshes located diagonally with respect to *Γ*_11_, including for *Γ*_23_, *Γ*_32_, and *Γ*_33_. The subnet *Γ*_1_ constituting a sum of *Γ_ij_* (for *i,j* = 1 to 3) is located between the planes (*x,z*) and (*y,z*) in the dihedral angle containing the positive axis *y* and the negative axis *x* (Figure 19). It is about one quarter of the designed resultant *z*-axis-symmetric *Γ* (Figure 16).

## 7. Discussion

The first basic goal accomplished by the proposed novel method is to create a relatively simple and regular spatial arrangement of many simple complete free forms connected to each other into one complex building free-form with oblique plane elevations and a transformed multi-segment shell roof structure. The individual free forms are roofed with single ruled shells whose shapes result from the expected shape transformations of thin-walled folded sheets. The simplicity of the method’s algorithm is to build a system of planes that, when intersected, isolate many spatial meshes creating a reference network adaptable to various boundary conditions. These complete meshes also define the facade walls of the designed complete free-forms. In these planes, common directrices of the shell segments roofing adjacent single free-forms are included. In the article, mutual skew straight lines are investigated as the roof directrices.

**Figure 20:**
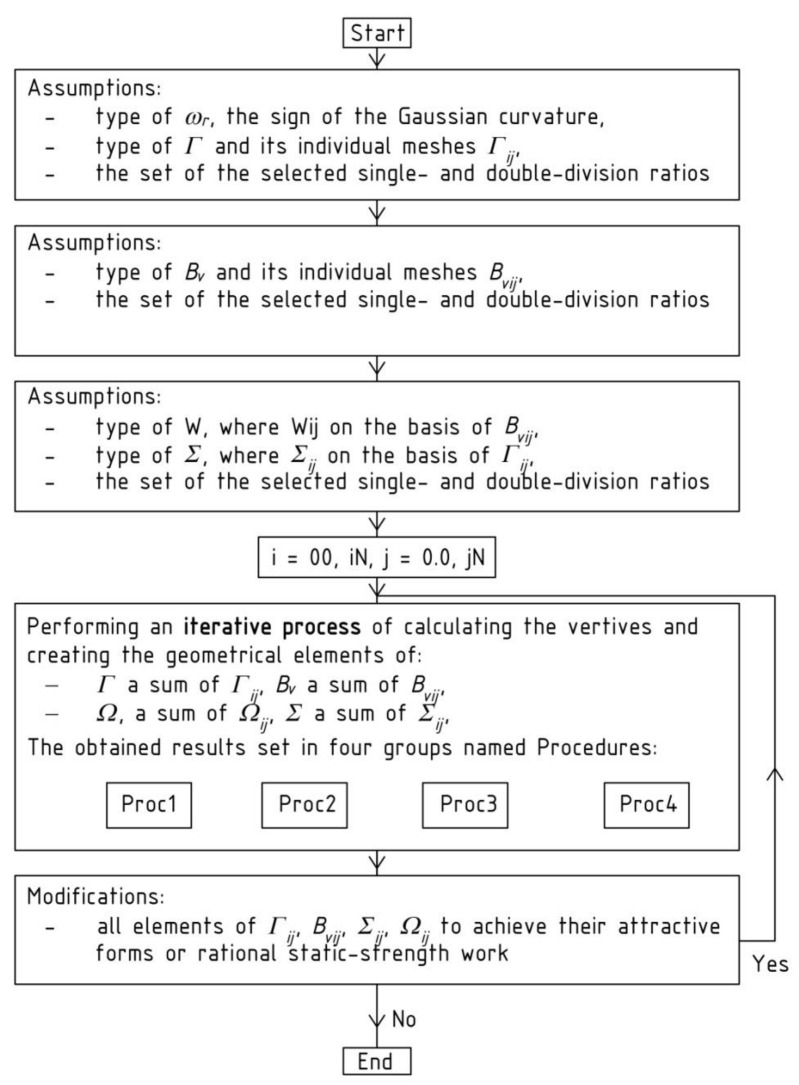
A block scheme of the method’s algorithm.

The regularity of numerous arrangements of many individual roof shell segments is ensured by the specific geometric shaping of these segments, based on the reference surface and division coefficients related to the intersecting point of respective planes and side edges of the reference networks. The most important steps of the method’s algorithm leading to the achievement of the above assumed goal by means of a parameterization based on the aforementioned division coefficients are presented in Figure 11 and Figure 20. The multitude and complexity of the activities and objects realized by the algorithm need to use computer technology to program the calculations and create the required geometric models.

The second main goal implemented by the method is the possibility of a relatively free and intuitive shaping of complex building free-form structures and their easy modification to the requirements and expectations of the designer.

To build a complex building form, the presented method’s algorithm instructs the iterative activities and objects presented by means of the block scheme shown in Figure 11. At the beginning of each *Γ*’s and *B_v_*’s shaping process, a set of numbers defining the locations of the vertices *W_ABij_*, *W_CDij_*, *W_ADij_* and *W_BCij_* of all subsequent reference tetrahedrons *Γ_ij_* of *Γ* is adopted. The numbers are the division coefficients of the proper pairs of the already constructed vertices belonging to *Γ_ij_* by the other sought-after vertices belonging to the subsequently determined tetrahedrons *Γ_i_*_+1*j*_, *Γ_ij_*_+1_ and *Γ_i_*_+1*j*+1_.

To create tetrahedrons *Γ*_1*j*_ and *Γ_i_*_1_ forming two principal orthogonal strips of *Γ*, a specific set of numbers constituting dependent or independent variables has to be adopted. To determine four vertices of each new reference tetrahedron located in one of these orthogonal strips, the following relations must be taken. Each tetrahedron has to have one common wall and two common side edges with one of the previously built reference tetrahedrons. Three other walls and two remaining side edges of this new reference tetrahedron are determined by means of two searched and two known vertices.

In order to determine the diagonal strips of the network *Γ*, a new set of parameters defining the location of four vertices of each tetrahedron *Γ_ij_* located diagonally has to be adopted, so that: (1) two its walls and three side edges have to be common with some tetrahedrons constructed previously, and (2) one of these side edges must also be common with one of the previously constructed tetrahedrons of the same diagonal strip. Based on the above set, the locations of the remaining fourth side edge and two walls of this diagonal reference tetrahedron, for example *Γ*_22_, are sought by means of two sought-after points belonging to two different side edges created previously.

Subsequently, the points *S_Aij_*, *S_Bij_*, *S_Cij_* and *S_Dij_* of a reference surface *ω_r_* and the vertices of all *B_vij_* meshes are determined on the basis of the reference network *Γ* and the adopted sets of initial data. These parameters are single-division coefficients and double-division coefficients of the subsequently selected pairs of the *Γ*’s vertices by: (1) the points *S_Aij_*, *S_Bij_*, *S_Cij_* and *S_Dij_*, (2) the vertices *A_ij_*, *B_ij_*, *C_ij_* and *D_ij_* of *B_vij_*, and (3) the points *P_Aij_*, *P_Bij_*, *P_Cij_* and *P_Dij_* of the base of the free-form-shaped building. In the example presented in the previous section, the calculated values of the division coefficients related to the positions of the aforementioned vertices are listed in Table 7.

**Figure 21:**
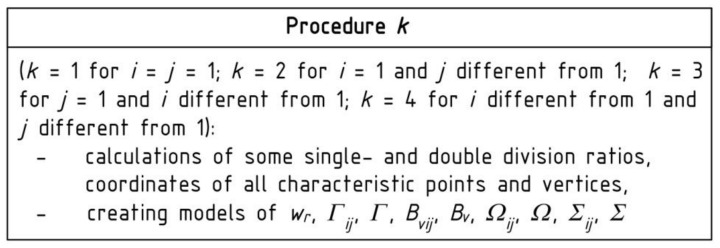
Common operations accomplished by the four configurations Prod*k*, where *k* = 1 to 4.

The main step of the method’s algorithm is accomplished by means of the activities assigned to the sections Proc*k*, where *k* = 1 to 3 (Figure 20) depending on the location of *Γ_ij_* in *Γ*. There are four configurations of these procedures, supporting the activities requiring different input data and various type and number of the functions used to determine the *Γ*’s and *B_v_*’s vertices. The common actions assigned to all configurations are presented in the container called Procedure *k* shown in Figure 21. The activities are implemented in the innovative computer application written in the AutoLISP programming language.

The actions of the section Proc1 (for *k* = 1) concern the first constructed meshes of networks *Γ* and *B_v_* and the sectors of structures *Ω* and *∑*, where *i* = *j* = 1. The activities referred to the section Proc2 (for *k* = 2), relating to the meshes of the first of two orthogonal strips of *Γ*, *B_v_*, *Ω* and *∑* running along the plane (*x,z*), for which *i* = 1 and *j* different from 1. The activities of the section Proc3 (for *k* = 3) relate to the meshes of the second orthogonal strip passing along the plane (*y,z*), for which *j* = 1 and *i* is different from 1. The activities assigned to the section Proc4 (for *k* = 4) refer to all meshes of the diagonal strips of *Γ*, *B_v_*, *Ω* and *∑*, for which *i* and *j* are different from 1. A novel computer-aided method is proposed due to the complexity of the above operations and geometric objects.

The division coefficients adopted initially to define the characteristic points of *Γ*, *ω_r_* and *B_v_* are also used for assessing the curvature of the reference surface *ω_r_* and the roughness of the eaves net *B_v_*. The expected curvature of the *ω_r_*’s area corresponding to the single mesh *Γ_ij_* can be shaped by means of four division coefficients (*W_ABij_*,*W_ADij_*)\*S_Aij_*, (*W_ABij_*,*W_BCij_*)\*S_Bij_*, (*W_CDij_*,*W_BCij_*)\*S_Cij_* and (*W_CDij_*,*W_ADij_*)\*S_Dij_* determining the positions of the points *S_Aij_*, *S_Bij_*, *S_Cij_* and *S_Dij_* on the side edges *a_ij_*, *b_ij_*, *c_ij_* and *d_ij_.* Since the points are created for a finite number of subsequent meshes *Γ_ij_*, then the curves located on *ω_r_* can be defined in an approximate way on the basis of these points. These curves also define *ω_r_* in an approximate way.

For the considered networks *Γ* and *B_v_* and structures *Ω* and *∑*, roughness of the *B_v_* network also plays an important role. The roughness is determined by means of division coefficients of the respective pairs of the *Γ*’s vertices by the selected *ω_r_*’s points and *B_v_*’s vertices. The roughness describes the disturbances in the smoothness of the network *B_v_* in relation to the smooth surface *ω_r_*. The disturbances result from the forced mutual inclination of each pair of the adjacent roof directrices designed for all complete meshes *B_vij_* to realize the shape transformations of the complete corrugated roof shells. On the one hand, the roughness can have a positive effect on the attractiveness and span of the complex roof free forms *Ω* and entire building *∑*. On the other hand, the edges between the adjacent meshes *B_vij_* can reduce the visual attractiveness of the building shaped by disturbing the smoothness of the shell roof rib structure.

The maximum and minimum values of the absolute and relative roughness of the entire network *B_v_* and the proportions between these values can be defined as double- and triple-division coefficients if the need exists. This problem also goes beyond the scope of the article.

**Figure 22:**
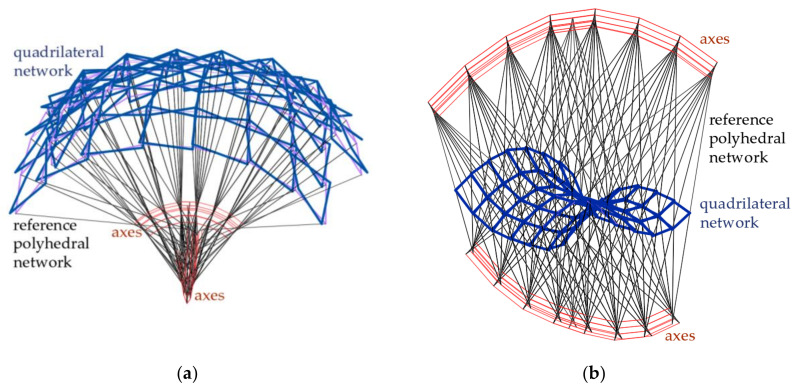
Two shell roof structures with eaves meshes arranged compatible with a regular surface of characterized by: (**a**) the positive Gaussian curvature, (**b**) the negative Gaussian curvature defined by means of two various polyhedral reference networks.

The division coefficients of the selected pairs of the *Γ_ij_*’s vertices by the points *S_Aij_*, *S_Bij_*, *S_Cij_* and *S_Dij_* affect the *ω_r_*’s curvature. If the values of the above coefficients are greater than 1, convex roof free forms with positive Gaussian curvatures are shaped (Figure 22a). If the values of the above coefficients are greater than 0 and less than 1, then concave roof shell forms with negative Gaussian curvature are shaped (Figure 22b). If the values of the above coefficients are from the range (-∞,0), concave roof free forms with positive Gaussian curvature are shaped. These forms have oblique facades tapering upwards in contrast to the forms widening upwards, e.g., the one presented in Figure 22a. A possibility of modifying the reference networks *Γ* by changing some division coefficients defining their shapes and the positions of their vertices allows one to optimize the curvature of *ω_r_* and the roughness of *B_v_* according to the demands referring to the attractiveness of the shaped building free-forms.

The third basic goal realized by means of the method is attractiveness and rationality of the final complex building free forms *∑*, which is associated with the properties of the investigated networks *Γ* and *B_v_*, and surface *ω_r_*. The main elements determining the attractiveness are: (1) the *ω_r_*’s curvature in two orthogonal and diagonal directions, (2) the positions of the *Γ*’s planes, and (3) the positions of the *B_v_*’s vertices on the *Γ*’s side edges with respect to *ω_r_*. Modifications of these elements enable us to maintain the expected shape and slope of the designed multi-plane facades and multi-segment shell roof structure, as well as their geometric coherence with the entire building free form.

The main elements determining the rationality of the investigated geometric structures are: (1) the mutual consistency and regular arrangement of all individual forms *∑_ij_*, (2) the mutual consistency and the regular arrangement of all individual roof forms *Ω_ij_*, (3) the consistency of the entire roof and facade forms, (4) the regularity of the distribution of all *Γ_ij_* relative to *ω_r_*, and (5) the location of the contraction of each effectively transformed fold along its length, induced by the mutual position and slope of the *B_vij_*’s and *Ω_ij_*’s directrices. The rationality is defined by means of the respective division coefficients or proportions between the selected elements of the investigated networks and structures.

## 8. Conclusions

The investigated innovative method’s algorithm utilizing the novel polyhedral reference networks for shaping unconventional visually attractive and shaped rational multi sector shell roofs, entire building free forms and their structural systems is proposed. The nominally plane thin-walled corrugated sheets transformed elastically and effectively into ruled shell shapes are the basic material impacting the shapes of the building elements.

The meshes of the reference networks enable the designer to define the eaves lines of all individual roof shell sectors made up of these sheets. The side edges and planes of these networks also define the elevations of the designed building free forms. Since the roof directrices of the complete roof shell sectors are skew straight or curved lines, it is convenient to contain them in the planes of the reference networks. Many innovative systems of such planes separating the roof shell sectors and containing the eaves lines including directrices of the shell sectors can simply and intuitively be developed as polyhedral reference networks *Γ* using computer technology, including the novel applications written for the CAD systems.

The investigated parameterization, regularity, symmetry and intuitiveness of the engineering computational models created with the help of the novel algorithm and its implementation in the novel computer applications enables one to shape attractive and rational building free-forms. The complex nature of the investigated building free forms and many interdisciplinary problems, as well as the proposed iterative diversified solutions of many complete issues, require the use of computer technology to obtain optimal solutions.

The attractiveness of the presented method results from the freedom of shaping of the diversified shell roof structures characterized by the positive, negative and zero Gaussian curvature and various patterns of many complete shell segments in the roof depending on the adopted values of the analyzed division coefficients related to the investigated reference networks *Γ* and eaves nets *B_v_*. Unlike other conventional methods, the extensive method for parametric shaping of the regular roof shell structures composed of many transformed corrugated shell sectors is developed based on the division coefficients. The regular shell units determined with *B_v_* are used as an orthotropic material for composing the innovative roof structures. In addition, the method’s algorithm takes account of the rectangular shapes of the folded sheets and the rational shape transformations of the shell units.

## Figures and Tables

**Table 1:** The coordinates of *Γ*_11_’s vertices.

Point	*x*-Coordinate (mm)	*y*-Coordinate (mm)	*z*-Coordinate (mm)
*W_AB_*_11_	−1000	0	0
*W_CD_*_11_	1000	0	0
*W_AD_*_11_	0	−871.6	9961.90
*W_BC_*_11_	0	871.6	9961.90

**Table 2:** The initial data defining the meshes *Γ*_11_ and *B_v_*_11_.

Division Coefficient	Value
(*W_AB_*_11_,*W_AD_*_11_)\*S_A_*_11_	5.5
(*W_AB_*_11_,*W_BC_*_11_)\*S_B_*_11_	5.5
(*W_CD_*_11_,*W_AD_*_11_)\*S_C_*_11_	5.5
(*W_CD_*_11_,*W_AD_*_11_)\*S_D_*_11_	5.5
(*W_AB_*_11_,*W_AD_*_11_)\(*S_A_*_11_,*A*_11_)	−0.1
(*W_AB_*_11_,*W_BC_*_11_)\(*S_B_*_11_,*B*_11_)	0.1
(*W_CD_*_11_,*W_BC_*_11_)\(*S_C_*_11_,*C*_11_)	−0.1
(*W_CD_*_11_, *W_AD_*_11_)\(*S_D_*_11_,*D*_11_)	0.1

**Table 3:** The coordinates of the *Γ*_12_’s vertices.

Point	*x*-Coordinate (mm)	*y*-Coordinate (mm)	*z*-Coordinate (mm)
*W_AB_*_12_	4500.00	4880.70	55,786.90
*W_CD_*_12_	3254.30	0	468.2
*W_AD_*_12_	−100	−958.7	10,958.10
*W_BC_*_12_	−100	958.7	10,958.10

**Table 4:** The data defining the mesh *B_v_*_12_.

Ratio	Value
(*W_AB_*_12_,*W_AD_*_12_)\*S_A_*_12_	5
(*W_AB_*_12_, *W_BC_*_12_)\*S_B_*_12_	5
(*W_CD_*_12_, *W_BC_*_12_)\*S_C_*_12_	5
(*W_CD_*_12_, *W_AD_*_12_)\*S_D_*_12_	5
(*W_AB_*_12_,*W_AD_*_12_)\(*S_A_*_12_,*A*_12_)	0.091
(*W_AB_*_12_, *W_BC_*_12_)\(*S_A_*_12_,*B*_12_)	−0.091
(*W_CD_*_12_, *W_BC_*_12_)\(*S_A_*_12_,*C*_12_)	0.091
(*W_CD_*_12_, *W_AD_*_12_)\(*S_A_*_12_,*D*_12_)	−0.091

**Table 5:** The coordinates of the *Γ*_22_’s vertices.

Point	*x*-Coordinate (mm)	*y*-Coordinate (mm)	*z*-Coordinate (mm)
*W_AB_*_22_	1100.00	−87.2	−996.2
*W_CD_*_22_	3589.70	−95.9	−580.8
*W_AD_*_22_	−100	958.7	10,958.10
*W_BC_*_22_	−110	2840.10	10,744.40

**Table 6:** The initial data defining the mesh *B_v_*_22_.

Ratio	Value
(*W_AB_*_22_,*W_AD_*_22_)\*S_A_*_22_	4.6667
(*W_AB_*_22_, *W_BC_*_22_)\*S_B_*_22_	4.6281
(*W_CD_*_22_, *W_AD_*_22_)\*S_D_*_22_	4.6667
(*W_CD_*_22_, *W_AD_*_22_)\*S_D_*_22_	4.6364
(*W_AB_*_22_,*W_AD_*_22_)\(*S_A_*_21_,*A*_22_)	−0.0833
(*W_AB_*_22_, *W_BC_*_22_)\(*S_A_*_21_,*B*_22_)	0.0826
(*W_CD_*_22_, *W_BC_*_22_)\(*S_A_*_22_,*C*_22_)	−0.0833
(*W_CD_*_22_, *W_AD_*_22_)\(*S_A_*_22_,*D*_22_)	0.0826

**Table 7:** The division coefficients defining selected *B_v__ij_*.

Ratio	Value
(*W_AB_*_11_,*W_AD_*_11_)\*A*_11_	5.4
(*W_AB_*_11_,*W_BC_*_11_)\*B*_11_	5.6
(*W_CD_*_11_,*W_BC_*_11_)\*C*_11_	5.4
(*W_CD_*_11_,*W_AD_*_11_)\*D*_11_	5.6
(*W_AB_*_12_,*W_AD_*_12_)\*A*_12_	5.091
(*W_AB_*_12_, *W_BC_*_12_)\*B*_12_	−4.909
(*W_CD_*_12_, *W_BC_*_12_)\*C*_12_	5.091
(*W_CD_*_12_, *W_AD_*_12_)\*D*_12_	−4.909
(*W_AB_*_22_,*W_AD_*_22_)\*A*_22_	4.5833
(*W_AB_*_22_, *W_BC_*_22_)\*B*_22_	4.7107
(*W_CD_*_22_, *W_BC_*_22_)\*C*_22_	4.5833
(*W_CD_*_22_, *W_AD_*_22_)\*D*_22_	4.7107

## Data Availability

Data is contained partially within the article and partially in the author’s article: Abramczyk, J. Symmetric Free Form Building Structures Arranged Regularly on Smooth Surfaces with Polyhedral Nets. *Symmetry* **2020**, *12*, 763.

## Appendix A

**Table A1:** The coordinates of the vertices *W_ABij_*, *W_CDij_*, *W_ADij_*, *W_BCij_* (for *i, j* = 1, 2, 3) of the polyhedral reference network *Γ*_1_.

Point	*x*-Coordinate (mm)	*y*-Coordinate (mm)	*z*-Coordinate (mm)
*W_AB_*_13_	3254.30	0	468.2
*W_CD_*_13_	5572.70	0	1487.4
*W_AD_*_13_	−435.4	−1054.60	12,007.10
*W_BC_*_13_	−435.4	1054.60	12,007.10
*W_AB_*_21_	−1100.00	−87.2	−996.2
*W_CD_*_21_	1100.00	−87.2	−996.2
*W_AD_*_21_	−4500.00	−4880.70	55,786.90
*W_BC_*_21_	0	2574.00	9677.1
*W_AB_*_31_	−1210.00	−353.3	−2063.50
*W_CD_*_31_	1210.00	353.3	−2063.50
*W_AD_*_31_	0	2574.00	9677.1
*W_BC_*_31_	0	4374.60	9074.60
*W_AB_*_23_	3589.70	−95.9	−580.8
*W_CD_*_23_	6173.60	−105.5	435.5
*W_AD_*_23_	−435.4	1054.60	12,007.10
*W_BC_*_23_	−480	3133.70	11,876.90
*W_AB_*_32_	1210.00	353.3	−2063.50
*W_CD_*_32_	3959.70	−389.5	−1713.3
*W_AD_*_32_	−110	2840.10	10,744.40
*W_BC_*_32_	−121	4847.40	10,188.40
*W_AB_*_33_	3959.70	−389.5	−1713.3
*W_CD_*_33_	6838.90	−429.4	−708.6
*W_AD_*_33_	−480	3133.70	11,876.90
*W_BC_*_33_	−529.1	5371.00	11,378.60

**Table A2:** The coordinates of the points *S_Aij_*, *S_Bij_*, *S_Cij_*, *S_Dij_* (for *i, j* = 1, 2, 3) of the reference surface network *ω*_*r*_.

Point	*x*-Coordinate (mm)	*y*-Coordinate (mm)	*z*-Coordinate (mm)
*S_A_*_11_	4500.00	−4793.60	54,790.70
*S_B_*_11_	4500.00	4880.70	55,786.90
*S_C_*_11_	−4500.00	4793.60	54,790.70
*S_D_*_11_	−4500.00	−4880.70	55,786.90
*S_A_*_12_	−4500.00	−4880.70	55,786.90
*S_B_*_12_	−4500.00	4793.60	54,790.70
*S_C_*_12_	−13,517.10	4793.60	52,918.00
*S_D_*_12_	−13,517.10	−4793.60	52,918.00
*S_A_*_13_	−13,517.10	−4793.60	52,918.00
*S_B_*_13_	−13,517.10	4793.60	52,918.00
*S_C_*_13_	−21,737.10	4793.60	49,304.20
*S_D_*_13_	−21,737.10	−4793.60	49,304.20
*S_A_*_21_	4500.00	4880.70	55,786.90
*S_B_*_21_	4500.00	13,460.50	53,340.40
*S_C_*_21_	−4500.00	13,460.50	53,340.40
*S_D_*_21_	−4500.00	4793.60	54,790.70
*S_A_*_31_	4500.00	13,460.50	53,340.40
*S_B_*_31_	4500.00	21,957.50	50,497.30
*S_C_*_31_	−4500.00	21,957.50	50,497.30
*S_D_*_31_	−4500.00	13,460.50	53,340.40
*S_A_*_22_	−4500.00	4793.60	54,790.70
*S_B_*_22_	*xS_C_*_21_	13,460.50	53,340.40
*S_C_*_22_	−13,675.60	13,605.30	52,270.20
*S_D_*_22_	−13,517.10	4793.60	52,918.00
*S_A_*_23_	−13,517.10	4793.60	52,918.00
*S_B_*_23_	−13,675.60	13,605.30	52,270.20
*S_C_*_23_	−22,104.00	13,660.90	49,061.50
*S_D_*_23_	−21,737.10	4793.60	49,304.20
*S_A_*_32_	−4500.00	13,460.50	53,340.40
*S_B_*_32_	−4500.00	21,957.50	50,497.30
*S_C_*_32_	−13,692.40	22,263.80	49,770.70
*S_D_*_32_	−13,675.60	13,605.30	52,270.20
*S_A_*_33_	−13,675.60	13,605.30	52,270.20
*S_B_*_33_	−13,692.40	22,263.80	49,770.70
*S_C_*_33_	−22,428.40	22,611.20	47,304.50
*S_D_*_33_	−22,104.00	13,660.90	49,061.50

**Table A3:** The coordinates of the vertices *A_ij_*, *B_ij_*, *C_ij_*, *D_ij_* (for *i, j* = 1, 2, 3) of the eaves edge net *B*_*v*1_.

Point	*x*-Coordinate (mm)	*y*-Coordinate (mm)	*z*-Coordinate (mm)
*A*_11_	4400	−4706.4	53,794.50
*B*_11_	4600	4880.7	55,786.90
*C*_11_	−4400.0	4706.4	53,794.50
*D*_11_	−4600.0	−4880.7	55,786.90
*A*_12_	−4390.0	−4697.7	53,694.90
*B*_12_	−4610.0	4889.4	55,886.50
*C*_12_	−13,517.1	4697.9	51,869.00
*D*_12_	−13,852.6	−4889.4	53,967.00
*A*_13_	−13,148.2	−4688.1	51,764.10
*B*_13_	−13,886.1	4899	54,071.90
*C*_13_	−21,136.3	4688.1	48,252.20
*D*_13_	−22,338.0	−4899.0	50,356.20
*A*_21_	4390	4697.7	53,694.90
*B*_21_	4610	13,726.60	54,407.70
*C*_21_	−4390.0	13,194.40	52,273.00
*D*_21_	−4610.0	4889.4	55,886.50
*A*_31_	4379	13,167.70	52,166.30
*B*_31_	4621	22,430.30	51,611.1
*C*_31_	−4379.0	21,484.70	49,383.50
*D*_31_	−4621.0	13,753.20	54,514.40
*A*_22_	−4400.0	4706.4	53,794.50
*B*_22_	−4621.0	13,753.20	54,514.40
*C*_22_	−13,367.3	13,360.70	51,326.40
*D*_22_	−13,886.1	4899	54,071.90
*A*_23_	−13,517.1	4697.9	51,869.00
*B*_23_	−13,983.9	13,850.00	53,213.90
*C*_23_	−21,549.5	13,391.00	48,108.10
*D*_23_	−22,338.0	4899	50,356.20
*A*_32_	−4390.0	13,194.40	52,273.00
*B*_32_	−4621.0	22,430.30	51,611.10
*C*_32_	−13,352.3	21,827.40	48,778.90
*D*_32_	−13,983.9	13,850.00	53,213.90
*A*_33_	−13,367.3	13,360.70	51,326.40
*B*_33_	−14,032.4	22,700.20	50,762.60
*C*_33_	−21,916.7	22,208.40	46,465.10
*D*_33_	−22,658.4	13,930.90	50,015.00
